# The Gut–Organ Axis within the Human Body: Gut Dysbiosis and the Role of Prebiotics

**DOI:** 10.3390/life13102023

**Published:** 2023-10-08

**Authors:** Georgia Saxami, Evangelia N. Kerezoudi, Christos Eliopoulos, Dimitrios Arapoglou, Adamantini Kyriacou

**Affiliations:** 1 Department of Nutrition and Dietetics, Harokopio University, 17671 Athens, Greece; evangelia.kerezoudi@oru.se (E.N.K.); akyriacou@hua.gr (A.K.); 2 School of Medical Sciences, Faculty of Medicine and Health, Örebro University, SE-701 82 Örebro, Sweden; 3 Institute of Technology of Agricultural Products, Hellenic Agricultural Organization—Demeter, L. Sof. Venizelou 1, 14123 Lykovryssi, Greece; chris_eliopoulos@hotmail.com (C.E.); dimarap@elgo.gr (D.A.)

**Keywords:** gut–organ axis, gut microbiota dysbiosis, prebiotics

## Abstract

The human gut microbiota (GM) is a complex microbial ecosystem that colonises the gastrointestinal tract (GIT) and is comprised of bacteria, viruses, fungi, and protozoa. The GM has a symbiotic relationship with its host that is fundamental for body homeostasis. The GM is not limited to the scope of the GIT, but there are bidirectional interactions between the GM and other organs, highlighting the concept of the “gut–organ axis”. Any deviation from the normal composition of the GM, termed ”microbial dysbiosis”, is implicated in the pathogenesis of various diseases. Only a few studies have demonstrated a relationship between GM modifications and disease phenotypes, and it is still unknown whether an altered GM contributes to a disease or simply reflects its status. Restoration of the GM with probiotics and prebiotics has been postulated, but evidence for the effects of prebiotics is limited. Prebiotics are substrates that are “selectively utilized by host microorganisms, conferring a health benefit”. This study highlights the bidirectional relationship between the gut and vital human organs and demonstrates the relationship between GM dysbiosis and the emergence of certain representative diseases. Finally, this article focuses on the potential of prebiotics as a target therapy to manipulate the GM and presents the gaps in the literature and research.

## 1. Introduction

The significance of the GM to human health has been recognised for centuries; Hippocrates said, “Death sits in the bowls” in 400 B.C., and the term “microbiota” dates back to the early 1900s [1]. The human GM is the largest micro-ecosystem in the human body and is regarded as the “essential organ” [2]. The GM is a complex, dynamic, and spatially heterogeneous ecosystem comprised of a collection of bacteria, viruses, fungi, and protozoa that colonise the gastrointestinal tract (GIT) and interact with each other and the human host [3]. The human body harbours a nearly equal quantity of microbial cells, in comparison to human cells [4]. The regions with the highest microbial biomass are the caecum and proximal colon.

The GM profile of each individual is unique at the species and genus level and is influenced by several factors, such as genetics, diet, environmental conditions, lifestyle, early microbial exposure, and the immune system [5]. However, the relative abundance and distribution at the phylum level along the intestine are consistent among healthy individuals [6]. The gut of an adult individual is majorly dominated by six phyla, including *Firmicutes* (*Clostridium*, *Lactobacillus*, and *Enterococcus*), *Bacteroidetes* (*Bacteroides*), *Actinobacteria* (*Bifidobacterium*), *Proteobacteria* (*E. coli*), *Fusobacteria*, *Verrucomicrobia*, and *Cyanobacteria*, among which *Firmicutes* and *Bacteroidetes* are the major types [7]. Also, fungi, mainly *Candida*, *Saccharomyces*, *Malassezia*, and *Cladosporium*, are included in the GM, as are viruses, phages, and archaea, mainly *Methanobrevibacter smithii* [8,9].

**Figure 1:**
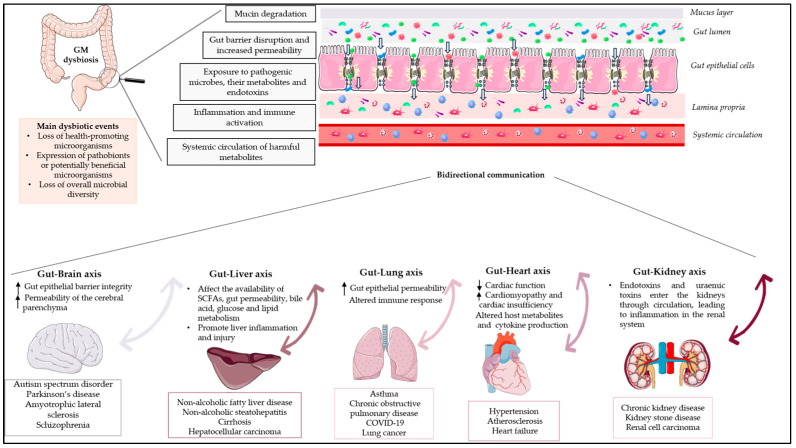
Schematic diagram depicting the influence of GM dysbiosis on the gut–organ axis. GM dysbiosis leads to the degradation of mucin, disrupts the gut’s protective barrier, increases its permeability, and enables pathogenic microorganisms, along with their by-products and endotoxins, to infiltrate. This invasion results in the activation of immune cells and triggers systemic inflammation through the peripheral circulation. The impact of GM dysbiosis extends beyond the gastrointestinal tract. Recent research indicates two-way interactions between the GM and various organs, emphasizing the idea of a “gut–organ axis”. This communication is facilitated through a range of signalling pathways and direct interactions between the host and the GM. Arrows indicate a bidirectional relationship between the gut and each organ. Parts of the figure were drawn using pictures from Servier Medical Art. Servier Medical Art by Servier is licensed under a Creative Commons Attribution 3.0 Unported License (https://creativecommons.org/licenses/by/3.0/ accessed on 25 August 2023).

The GM has a symbiotic relationship with the host, while it has a central role in maintaining the homeostasis of the human body, impacting various physiological functions, including metabolism, vitamin synthesis, barrier homeostasis, protection against pathogens, immune system development and maturation, and hematopoiesis via intestinal and extra-intestinal actions, having an effect on human behaviour and thereby making it a vital organ [3,10]. The influence of the GM is not limited to the scope of the GIT, but evidence from recent studies describes bidirectional interactions between the GM and other organs, highlighting the concept of the “gut–organ axis” (Figure 1). This cross-talk is mediated by a variety of signalling pathways and direct chemical interactions between the host and microorganisms [10]. Studies over the past five years have increased our understanding of the gut–brain axis, the gut–liver axis, the gut–lung axis, and the gut–heart axis [11].

Any deviation from the normal composition of the GM, termed “microbial dysbiosis”, is characterised by an imbalance in the composition and/or function of the microbial ecology. Dysbiosis has been classified into numerous types or combinations of types, including (1) the loss of health-promoting microorganisms; (2) the expression of pathobionts or potentially beneficial microorganisms; and (3) the loss of overall microbial diversity (Figure 1) [12]. Environmental factors as well as host-related factors can influence homeostasis, such as perinatal disruption of colonization, genetics, diet, disease, and stress [13]. Several studies have highlighted the dysbiosis of the GM during the course of diseases such as inflammatory bowel disease (IBD), malnutrition, metabolic disorders, asthma, and neurodegenerative diseases. In most diseases, it has been reported that altered microbiota causes pathophysiologies in vital human organs; however, few studies have demonstrated the causal relationship between microbial alterations and disease phenotypes, and it remains unclear whether the altered GM contributes to a disease or simply reflects its status [13].

According to the International Scientific Association for Probiotics and Prebiotics (ISAPP), prebiotics are substrates that are “selectively utilized by host microorganisms, conferring a health benefit” [14]. Fructo-oligosaccharides (FOS), galacto-oligosaccharides (GOS), lactulose, and inulin are the most widely recognised prebiotics, whereas β-glucans derived from various mushroom species (e.g., *Pleurotus eryngii*) are potential prebiotic candidates [15]. Recently, whole-food-based treatments have been used to modulate the GM through potential synergistic interactions between food’s various components [15]. Restoration of the GM in various diseases with pro/prebiotics has been postulated, but evidence for the effects of prebiotics is scarce.

In this review, we examine and discuss the bidirectional relationship between and key characteristics of the gut and vital human organs in the context of dysbiosis. Additionally, we investigate the link between GM dysbiosis and the development of specific representative diseases. Finally, this article emphasises the role of prebiotics and their significance in the restoration of the dysbiotic gut, while also highlighting the gaps in the existing literature and research on prebiotics.

## 2. The Gut–Brain Axis

The gut–brain axis comprises a complex physiological system that enables bidirectional communication between the gut and the host nervous system. [16]. This bidirectional communication within the gut–brain axis elucidates how messages from the GM influence brain function and how signals from the brain impact gastrointestinal physiology and gut microbial activity [17]. These bidirectional communications involve the central nervous system (CNS), intrinsic branches of the enteric nervous system (ENS), extrinsic parasympathetic and sympathetic branches of the autonomic nervous system (ANS), the hypothalamic–pituitary–adrenal axis (HPA), neuroimmune pathways (neurotransmitters, hormones, and neuropeptides), and the gut microenvironment [15,18]. The HPA axis, a component of the limbic system, is considered the central stress efferent axis that coordinates the organism’s adaptive responses to all stressors. Environmental stress and elevated systemic pro-inflammatory cytokines activate this system, which, via the secretion of the corticotropin-releasing factor from the hypothalamus, stimulates adrenocorticotropic hormone secretion from the pituitary gland, which ultimately results in cortisol release from the adrenal glands [6]. Thus, the combination of neural and hormonal lines of communication allows the brain to influence the activities of gut functional effector cells, including immune cells, epithelial cells, and enteric neurons [19]. On the other hand, these same cells are influenced by the GM, which may influence these central processes directly and indirectly via immune system activation, the production of neurotransmitters, and the production of short-chain fatty acids (SCFAs) and key dietary amino acids such as tryptophan and its metabolites [20]. Furthermore, the GM can act through the permeability of the gut barrier, with an increase in circulating lipopolysaccharide (LPS), modulating the levels of brain-derived neurotrophic factor and altering neuroendocrine and neural pathways.

In addition, the brain affects gut peristalsis, and sensory and secretion function, mainly via the vagus nerve. The vagus nerve, which transmits information from the luminal environment to the CNS, is the major nerve of the parasympathetic system of the ANS and a crucial modulatory constitutive direct communication pathway between the GM and the brain [21]. The vagus nerve consists of sensory and motor neurons and has been extensively studied for its involvement in hunger, satiety, and stress response but also for its major role in the regulation of inflammation via neuronal motor efferents [22].

The gut–brain axis is expected to have many effects on mood, motivation, and higher cognitive functions, in addition to ensuring that gastrointestinal homeostasis is properly maintained [6]. Disruption of the delicate balance between host and gut bacteria could be a contributing factor behind various diseases. The dysregulation of the gut–brain axis has been linked by numerous researchers to various immunologic, neurologic, and psychiatric disorders.

### 2.1. Gut Dysbiosis in Neurologic Diseases

GM dysbiosis interferes with the development of local and systemic inflammatory states, resulting in altered gut epithelial barrier integrity, allowing the release of hormones, microbial metabolites, and components by the GM that reach the brain via the vagus nerve, crossing the blood–brain barrier, and inducing neurodegenerative processes [23]. Moreover, dysbiosis increases the permeability of the cerebral parenchyma, which may result in neuroinflammation and dysfunctional neuronal cells. Emerging research indicates that gut dysbiosis may influence the onset and progression of a variety of neurological disorders, such as autism spectrum disorder (ASD), Parkinson’s disease (PD), amyotrophic lateral sclerosis (ALS), and schizophrenia. Table 1 provides a summary of the main dysbiotic events on the GM composition identified in neurological disorders. Subsequent sections will delve into the analysis of representative diseases and their associated dysbiotic events.

#### 2.1.1. Dysbiosis in Autism Spectrum Disorder

Autism spectrum disorder (ASD) is a complex group of neurodevelopmental disorders characterised by aberrant social interactions and communication, repetitive and stereotyped patterns of behaviour, and abnormal sensory responses. [24]. According to a recent systematic literature review, the prevalence of ASD in US children ranked 1.70 and 1.85% in children aged 4 and 8 years, respectively, while the prevalence in Europe ranged between 0.38 and 1.55% [25]. Although genetic and environmental factors have been linked to the development of ASD, the precise etiology remains unknown. Recent research has highlighted the role of the gut–brain axis in various neuropsychiatric disorders, including autism spectrum disorder. In addition, individuals with ASD frequently experience gastrointestinal disturbances, such as constipation, diarrhoea, flatulence, increased gut permeability, and abdominal pain [26,27].

Several studies have highlighted differences in the GM composition between ASD and neurotypical children [28]. It should be noted, however, that among studies related to ASD, no specific microbial species has been found to be significantly different, as various factors such as diet, age, sex, population, and severity of autism should be taken into account [28]. Although changes in the GM composition of autistic children are not always consistent across studies, patients frequently exhibit microbial imbalances of multiple types, including higher abundances of *Bacteroides*, *Parabacteroides*, *Clostridium*, *Faecalibacterium*, and *Phascolarctobacterium* and a lower relative abundance of *Streptococcus* and *Bifidobacterium* [26,29]. Recently, our research group demonstrated that neurotypical children exhibited increased levels of *Prevotella* spp. and *Bifidobacterium* spp. compared to ASD children [15]. In the same pattern, the study of Ding et al. [30] demonstrated that children with ASD showed an altered GM structure compared with children in the healthy control group. In addition, increased levels of unidentified *Lachnospiraceae*, *Clostridiales*, *Erysipelotrichaceae*, *Dorea*, *Collinsella*, and *Lachnoclostridium* strains and significantly lower levels of Bacteroides, *Faecalibacterium*, *Parasutterella*, and *Paraprevotella* were found in the ASD group compared with healthy children [30]. Interestingly, the structure of the GM community was associated with the severity of autistic symptoms, and the authors suggested that GM regulation may be a new strategy for ASD treatment in the future.

The gastrointestinal symptoms of individuals with ASD seem to be significantly correlated with the degree of behavioural and cognitive impairment. For example, in individuals with ASD, irritability, aggressiveness, sleep disturbances, and self-injury are strongly associated with GI symptoms [26,31]. This evidence suggests that gastrointestinal abnormalities, perhaps linked to gut dysbiosis, may be associated with ASD [32]. Consistent with this hypothesis, a meta-analysis by Iglesias-Vázquez et al. [29] suggests that there is a dysbiosis in ASD children that may influence the development and severity of ASD symptomatology. More specifically, this study concluded that the microbiota of ASD individuals was mainly composed of the phyla Bacteroidetes, Firmicutes, and Actinobacteria and also showed a significantly higher abundance of the genera *Bacteroides*, *Parabacteroides*, *Clostridium*, *Faecalibacterium*, and *Phascolarctobacterium* and a lower percentage of *Coprococcus* and *Bifidobacterium*. Taken together, all these alterations in the GM could be associated with increased GI disturbances in individuals with ASD.

#### 2.1.2. Dysbiosis in Parkinson’s Disease

Parkinson’s disease (PD) is the second most common degenerative disorder of the brain, affecting seven to ten million people worldwide [33]. PD is mainly characterised by multifactorial motor and non-motor symptoms, including resting tremor, muscular rigidity, slowness of movement, and gait abnormality, as well as cognitive disturbances, depression, mood deflection, sensory alternations, and sleep alternations [33,34]. The principal pathology of PD is characterised by the loss of dopamine-producing neurons present in a specific region of the brain, known as the substantia nigra, accompanied by the accumulation of alfa-synuclein (alfa-syn) in the form of Lewy bodies and Lewy neurites, a condition known as synucleinopathy [35].

Complex genetic and environmental factors are involved in the etiology of PD; however, the cause of PD remains unknown. Gastrointestinal symptoms are observed in most PD patients, including hypersalivation, dysphagia, constipation, nausea, altered bowel habits, and defecatory dysfunction [33]. Several studies have demonstrated GM abnormalities in patients with PD [36,37,38]. A meta-analysis conducted by Romano et al. [39] re-analysing the ten currently available 16S microbiome datasets found significant alterations in the PD-associated microbiome. More specifically, the authors concluded that enrichment of the genera *Lactobacillus*, *Akkermansia*, and *Bifidobacterium* and depletion of bacteria belonging to the *Lachnospiraceae* family and the *Faecalibacterium* genus, emerged as the most consistent PD gut microbiome changes, suggesting that the observed dysbiosis may be a result of pro-inflammation, which could be linked to the GI symptom manifestation in PD patients [39]. In another study, consistent increases were principally shown in the family *Verrucomicrobiaceae*, genus *Akkermansia*, and species *Akkermansia muciniphila*, while health-promoting genera and butyrate producers *Roseburia* and *Faecalibaterium* were reported to decrease in PD patients [40]. Emerging studies have shown the correlations between GM alterations and the phenotypes of PD, including both motor and non-motor symptoms [41,42,43]. These alterations in the GM of patients may reveal a mechanism, as this observed dysbiosis has been associated with increased intestinal barrier permeability and subsequent gut inflammation. This hypothesis is supported by a number of studies that demonstrate that GM dysbiosis in PD is shown to be associated with the disrupted intestinal barrier, which is closely associated with gut inflammation, an established symptom in PD patients [44,45].

#### 2.1.3. Dysbiosis in Amyotrophic Lateral Sclerosis (ALS)

Amyotrophic lateral sclerosis (ALS) is a fatal neurodegenerative disease defined by progressive loss of cortical, brain stem, and spinal motor neurons, resulting in weakness and wasting of the musculature [46,47]. In addition, ALS presents extra-motor features, including cognitive and behavioural disturbances [48]. Over 90% of ALS cases are sporadic (sALS) and of unknown cause, while the remaining 10% are familial (fALS) since they carry a mutation in one of the disease-related genes [48]. Mutations of superoxide dismutase 1 (SOD1), FUS RNA binding protein (FUS/TLS), C9orf72-SMCR8 complex subunit (C9orf72), and TAR DNA binding protein (TARDBP/TDP-43) are more commonly associated with ALS [49].

ALS etiology and pathophysiology require further elucidation, and in spite of massive efforts having been invested, there is no cure available at present, leading to death by respiratory failure within 2–5 years from symptom onset [50]. Recent studies demonstrate a strong pathophysiological crosstalk between the GM and ALS [51]. ALS pathogenesis has been linked to alterations in GM composition, impaired metabolism, an altered innate immune response, and the production of gut-derived neurotoxins by Clostridia species that induce brain damage [51].

Due to a number of factors, such as the small sample size, the observed heterogeneity within the study population, the various experimental procedures and data analysis, and the heterogeneity of the GM regardless of health status, the results of human studies conducted to determine the potential role of the GM in ALS patients are frequently inconclusive. Despite the contradictory results among the studies, we could observe some important findings, which include the following: (1) Differences in the GM populations between ALS patients and healthy individuals. For example, in the study of Fang et al. [52], which examined six ALS patients and five healthy people without ALS, the authors demonstrated significant differences in GM composition between the two groups. More specifically, in the gut of ALS patients, a reduced ratio of Firmicutes/Bacteroidetes was accompanied by a decreased abundance of butyrate-producing *Oscillibacter*, *Anaerostipes*, and *Lachnospira* counts and an increased abundance of glucose-metabolizing Dorea. More recently, comparing the GM of 10 ALS patients and their spouses (n = 10), it was found that the populations of the ALS patients’ GM were more diverse and deficient in *Prevotella* spp., suggesting that modifying the gut microbiome, such as via amelioration of *Prevotella* spp. deficiency, and/or altering butyrate metabolism, may have translational value for ALS treatment [53]. (2) GM composition alters during the course of the ALS. Gioia and colleagues [54] studied the GM of 50 ALS patients and 50 matched controls and demonstrated that the GM of ALS patients differed from that of controls. Also, the composition of the intestinal microbiota changed as the disease progressed, as indicated by a significant decrease in the number of operational taxonomy units observed during the follow-up. Intriguingly, an imbalance between potentially protective microbial groups, such as Bacteroidetes, and those with potential neurotoxic or pro-inflammatory activity, such as Cyanobacteria, has been observed.

Overall, these findings indicate the implication of the GM in ALS disease; however, it has been difficult to ascertain whether these changes in the GM are the cause of ALS, an aggravating factor for the disease, or the result of the disease. Additional human clinical research evidence is required in order to establish the exact role of the GM in the pathogenesis of ALS.

#### 2.1.4. Dysbiosis in Schizophrenia

Schizophrenia is a complex, heterogeneous, neurodevelopmental disorder with deficits across many dimensions [55]. The expression of the underlying genetic vulnerability is shaped by a multifaceted combination of prenatal and early postnatal environmental factors [56,57]. These factors may sensitise a developing brain and its information processing ability to the subsequent accumulation of additional environmental insults, which may overwhelm compensatory capacities during adolescence and emerge as psychotic symptoms [58]. Subtle deficits in cognition, social communication, and functioning are often evident prior to the onset of overt psychotic symptoms [59], and the majority of people experience recurring psychotic relapses with variable degrees of functional impairment [60].

A precise integrative mechanistic understanding of the interaction of genetic and environmental processes across the neurodevelopmental trajectory in this condition remains elusive. The link between schizophrenia and the GM has garnered increasing attention in recent years. The main findings of existing studies examining the link between the GM and schizophrenia include the following: 

(a) Patients with schizophrenia have a deviant GM compared to healthy controls. The diversity and composition of the GM were substantially altered in schizophrenia patients, according to these findings [61,62]. Zheng et al. [61] found significant alterations in beta diversity but not alpha diversity between the GM of patients and controls. In the schizophrenia group, an enhanced count of bacterial families like *Prevotellaceae*, *Veillonellaceae*, *Bacteroidaceae*, and *Coriobacteriaceae* was observed compared to healthy controls, while *Ruminococcus* and *Roseburia* abundances were significantly lower in patients with schizophrenia.

(b) Specific bacteria may function as biomarkers to differentiate patients with schizophrenia from healthy individuals [63,64]. Shen et al. identified 12 biomarkers that could be used as diagnostic factors to differentiate the schizophrenia cohort from the control cohort, including *Gammaproteobacteria* (at class level), *Enterobacteriales* (at order level), *Alcaligenaceae*, *Enterobacteriaceae*, and *Lachnospiraceae* (at family level), *Acidaminococcus*, *Phascolarctobacterium*, *Blautia*, *Desulfovibrio*, and *Megasphaera* (at genus level), and *Plebeius fragilis* (at species level).

(c) Differences in the GM between remission and acute schizophrenia. Pan et al. [64] demonstrated differences between acute and remission patients, indicating that alterations in the intestinal microbiota may influence the prognosis of the disease and suggesting the GM’s potential as a non-invasive diagnostic tool.

(d) Differences in the GM between first-episode drug-naïve and chronically medicated schizophrenia patients [65]. Chronically antipsychotic-treated schizophrenia patients showed lower microbial richness and diversity as compared to first-episode drug-naïve schizophrenia patients and healthy controls, suggesting that the gut microbiome may be implicated in the pathophysiology of schizophrenia via modulation of specific brain structures [65].

(e) The role of the gut–brain axis. The GM was found to be associated with schizophrenia via processes involved in the gut–brain axis, including immune-regulating pathways, neurotransmitter synthesis, the production of bioactive microbial metabolites, and tryptophan metabolism [66]. Schizophrenia-related behaviour has been observed in mice by Zheng et al. [61], who demonstrated that transplantation of the GM from schizophrenia patients induces schizophrenia-like behaviours in germ-free recipient rodents, suggesting that the GM can affect the brain neurochemistry associated with the onset of schizophrenia.

### 2.2. The Role of Prebiotics in Neurological Diseases

In recent years, different studies, including mostly in vitro and in vivo studies, and only a few human studies, have shown the beneficial effects of prebiotics on brain function [67,68]. The proposed mechanisms for prebiotic-based modulation of the GM–brain axis include the following [69,70,71]: (i) decreased inflammation in gut inflammatory disorders, preventing the presence of inflammatory compounds in the brain; (ii) improvement of GM composition and modulation of brain function, enhancing the composition of the GM; and (iii) influence on the production of neurochemicals. In addition, it has been suggested that, compared to probiotics, prebiotics could be advantageous due to probiotics’ inability to survive in the GI tract [69].

Numerous clinical studies examine the impact of probiotics and symbiotics on neurological conditions [72,73,74]. On the other hand, the supplementation of prebiotics to manipulate the GM as a novel treatment for neurological diseases has not been investigated, and there are only a few human studies that examine the effectiveness of prebiotics, while in ALS there have been no clinical studies (Table 2). The first study to examine the effects of prebiotics on ASD was conducted by Grimaldi et al. [75]. More particularly, the authors assessed the impact of a prebiotic (B-GOS^®^ mixture, Clasado Biosciences Ltd., Reading, UK) on GM composition and metabolic activity in 30 autistic children. According to the results, the administration of B-GOS led to modulation of the GM composition in autistic children following unrestricted diets. This modulation primarily affected bifidobacterial populations and also affected other bacterial groups, including members of the *Lachnospiraceae* family such as *Coprococcus* spp., *Dorea formicigenerans*, and *Oribacterium* spp. [75]. Furthermore, another study noted an amelioration of GM dysbiosis in children with ASD [76]. Dietary supplementation with partially hydrolysed guar gum (PHGG) in ASD children increased the relative prevalence of *Acidaminococcus* and *Blautia*, whereas the relative prevalence of *Streptococcus*, *Odoribacter*, and *Eubacterium* decreased. Also, prebiotic intervention decreased the behavioural irritability of ASD children [76]. Two studies have been conducted examining the effect of prebiotic supplementation with a simultaneous effect on GM modulation in Parkinson’s disease [77,78]. In the study of Becker et al. [78], an 8-week prebiotic intervention with resistant starch (RS) was conducted, enrolling 87 subjects distributed across three study arms: 32 PD patients who received RS, 30 control subjects who also received RS, and 25 PD patients who were provided with dietary instructions only. According to the results, a reduction in non-motor symptom load and a stable gut microbiome in PD patients after RS intervention were observed. In the study of Hall et al. [77], an open-label, non-randomised study was conducted in 10 newly diagnosed and 10 non-medicated and treated PD participants, wherein the impact of 10 days of prebiotic (bar containing resistant starch and rice brain) intervention was evaluated. The prebiotic supplementation resulted in a reduction in the relative abundance of potentially pro-inflammatory bacteria, such as Proteobacteria and Escherichia coli, while increasing the relative abundance of SCFA-producing bacteria, including *Faecalibacterium prausnitzii*. In addition, the unified Parkinson’s disease rating scale improved with prebiotic treatment [77]. The effects of prebiotic supplementation on schizophrenia were studied by Ido et al. [79]. More specifically, a female subject with schizophrenia was administered a prebiotic preparation of lactosucrose while keeping her medication unchanged. According to the results, after three months of lactosucrose administration, there was an improvement in psychotic symptoms, a significant decrease in the abundance of Clostridium, and an increased Bifidobacterium-to-Clostridium ratio [79].

More research is required to determine the effects of prebiotics in the management of neurological diseases. While there have been promising studies suggesting potential benefits, more comprehensive and long-term human research is needed to establish conclusive evidence.

**Table 1:** Main dysbiotic events that occur in GM during the onset and progression of neurological disorders.

Neurodegenerative Disease	Main Dysbiotic Events in GM	Reference
Autism spectrum disorder (ASD)	- Higher abundances of Bacteroides, Parabacteroides, Clostridium, Faecalibacterium, and Phascolarctobacterium and a lower relative abundance of Streptococcus and Bifidobacterium in ASD patients - Dysbiosis in ASD children may influence the development and severity of ASD symptomatology	[15,26,29]
Parkinson’s disease (PD)	- Enrichment of the genera *Lactobacillus*, *Akkermansia*, and *Bifidobacterium* and depletion of bacteria belonging to the *Lachnospiraceae* family and the *Faecalibacterium* genus in PD patients	[39,40]
Amyotrophic lateral sclerosis (ALS)	- Reduced ratio of *Firmicutes*/*Bacteroidetes*, decreased abundance of butyrate-producing *Oscillibacter*, *Anaerostipes*, *Lachnospira* counts, and *Prevotella*, increased abundance of glucose-metabolizing *Dorea* in ALS patients - GM composition alters during the course of the ALS	[52,54]
Schizophrenia	- In the schizophrenia group, an enhanced count of bacterial families like *Prevotellaceae*, *Veillonellaceae*, *Bacteroidaceae*, and *Coriobacteriaceae* was observed compared to healthy controls, while *Ruminococcus* and *Roseburia* abundances were significantly lower in patients with schizophrenia - Specific bacteria may function as biomarkers to differentiate schizophrenia from healthy individuals - Differences in GM between remission and acute schizophrenia as well as between first-episode drug-naïve and chronically medicated schizophrenia patients	[61,63,64,65]

**Table 2:** GM manipulation-based interventions with prebiotics in human health.

Disease	Study Design	Population	Prebiotic Compound	Effects on the Disease	Beneficial Effects on GM	Reference
Neurological diseases	Randomised, double-blind, placebo-controlled study	30 children diagnosed with ASD were categorised into two groups, A and B, based on their dietary habits. Group A consisted of children with unrestricted diets (n = 18), while Group B comprised those following an exclusion diet (n = 12). Subsequently, within each of these groups, children were assigned randomly to two feeding subgroups using a random number system. Group I received a placebo, while Group II was administered B-GOS^®^	B-GOS^®^ mixture (Bimuno^®^; Clasado Biosciences Ltd., Reading, UK) 1.8 g: 80% GOS content for a 6-week feeding period	Improvement in social behaviour scores	The administration of B-GOS led to modulation of the GM composition in autistic children following unrestricted diets. This modulation primarily affected bifidobacterial populations and also influenced other bacterial groups, including members of the *Lachnospiraceae* family such as *Coprococcus* spp., *Dorea formicigenerans*, and *Oribacterium* spp.	[75]
	Cohort study	13 ASD children aged 4–9 years	Partially hydrolysed guar gum (6 g/day) for two months or longer	Decrease the behavioural irritability	The relative prevalence of *Acidaminococcus* and *Blautia* increased, whereas the relative prevalence of *Streptococcus*, *Odoribacter*, and *Eubacterium* decreased	[76]
	Open-label, non-randomised study	20 participants with PD, consisting of 10 newly diagnosed, non-medicated individuals with PD and 10 individuals who were already receiving treatment for PD	Prebiotics in the form of a bar containing resistant starch, rice bran, resistant maltodextrin, and inulin for 10 days (one bar = 10 g fibre)	Unified Parkinson’s Disease Rating Scale improved with treatment	The consumption of prebiotics resulted in a reduction in the relative abundance of potentially pro-inflammatory bacteria, such as *Proteobacteria* and *Escherichia coli*, while increasing the relative abundance of bacteria known to produce SCFAs, including *Faecalibacterium prausnitzii*	[77]
	Monocentric, prospective, open-label clinical trial	The study included 87 subjects distributed across three study arms: 32 PD patients who received resistant starch, 30 control subjects who also received resistant starch, and 25 PD patients who were provided with dietary instructions only	5 g of resistant starch twice per day orally over a period of 8 weeks	Reduction in non-motor symptom load in the PD patients who received resistant starch	Stabilised faecal microbial diversity	[78]
		1 female subject with schizophrenia	A prebiotic preparation of lactosucrose (OligoOne^®^) 3.0 g/day was administered, with the medication unchanged	Improvement of psychotic symptoms	After three months of lactosucrose administration, there was a significant decrease in the abundance of *Clostridium* and an increased *Bifidobacterium* to *Clostridium* ratio. Additionally, improvements were observed in bowel movements, and there was a reduction in constipation	[79]
Liver diseases	Placebo-controlled, randomised pilot trial	14 individuals with liver-biopsy-confirmed NASH	The subjects were randomised to receive oligofructose (8 g/day for 12 weeks followed by 16 g/day for 24 weeks) or isocaloric placebo for 9 months	Prebiotic improved liver steatosis relative to placebo and improved overall NAS score	Oligofructose supplementation led to an increase in *Bifidobacterium* levels, while it resulted in a reduction of bacteria belonging to *Clostridium* cluster XI and I	[80]
	Small cohort single-centre study	Twenty-four subjects with histologically confirmed liver cirrhosis and a body mass index (BMI) of 25.78 kg/m^2^ were compared to 29 healthy controls	In the patient group, lactitol was administered orally at a dosage of 5 g three times daily, and samples were collected after four weeks of treatment	All clinical parameters, including MELD, showed no difference between pre- and post-lactitol treatment groups	After the lactitol intervention, there was an increase in the levels of health-promoting lactic acid bacteria, such as *Bifidobacterium longum*, B. *pseudo-catenulatum*, and *Lactobacillus salivarius*. Additionally, there was a significant decrease in the pathogen *Klebsiella pneumonia* and the associated antibiotic-resistant genes and virulence factors	[81]
Heart diseases	Randomised, placebo-controlled, double-blind cross-over trial	Untreated individuals with hypertension, being of either sex, 18–70 years of age, and having a BMI of 18.5–35 kg/m^2^	Participants were initially assigned to either Diet A or Diet B for a duration of 3 weeks. Diet A included HAMSAB (prebiotic acetylated and butyrylated high amylose maize starch) administered at a daily dosage of 40 g, while Diet B consisted of a daily intake of 40 g of a placebo over the same 3-week period. After a 3-week washout period, participants switched to the opposite diet arm for another 3 weeks	Reduction in ambulatory systolic blood pressure	HAMSAB intervention promoted the growth of the commensal bacteria *P. distasonis* and *R. gauvreauii* and supported the restoration of local production of SCFAs by these microbes	[82]
Kidney diseases	Double-blind, parallel, randomised, placebo-controlled trial	20 patients with end-stage CKD undergoing haemodialysis	The participants were randomised to two groups: one received biscuits containing 20 g/d of high-amylose maize-resistant starch type 2 (HAM-RS2), an insoluble, fermentable fibre, while the other received regular wheat flour (placebo) for the first month and 25 g/d during the second month	Decrease in in systemic inflammation (serum urea, IL-6, TNFα, and malondialdehyde)	Supplementation of amylose-resistant starch, HAM-RS2, in patients with CKD led to an increase in *Faecalibacterium*	[83]
	Randomised controlled clinical trial	32 patients with CKD in stages 3 and 4 were recruited and randomly assigned to intervention (n = 16) and control (n = 16) groups	Patients in intervention group received 30 mm lactulose syrup three timesa day for an 8-week period. Control group received placebo 30 mm three times a day	Creatinine significantly decreased in intervention group	Lactulose administration increase faecal *Bifidobacteria* and *Lactobacillus* counts in CKD patients	[84]
	Randomised, double-blind, placebo-controlled, crossover study	12 patients undergoing haemodialysis	Patients were randomised to consume inulin (10 g/d for females; 15 g/d for males) or maltodextrin (6 g/d for females; 9 g/d for males) for 4 weeks, with a 4-week washout period	Inulin did not reduce faecal p-cresol or indoles, or plasma concentrations of p-cresyl sulphate or indoxyl sulphate	Inulin increased the relative abundance of the phylum *Verrucomicrobia* and its genus *Akkermansia*. In addition, inulin and maltodextrin resulted in an increased relative abundance of the phylum *Bacteroidetes* and its genus *Bacteroides*	[85]
	Randomised single-centre, single-blinded control trial	59 predialysis participants with CKD in stages 3 to 5 were randomised	59 participants were randomised to either the β-glucan prebiotic intervention group (13.5 g of β-glucan prebiotic fibre supplement containing 6 g of fibre, of which 3 g was β-glucan per serving) daily (n = 30) or the control group (n = 29) for 14 weeks	Supplementation of β-glucan fibre resulted in reduced plasma levels of the free fraction of colon-derived uremic toxins, without a change in kidney function over the 14-week study period	High prevalence of *Bacteroides* 2 in the CKD population	[86]

## 3. The Gut–Liver Axis

The gut–liver axis represents one of the most important links between the GM and extra-intestinal organs. The gut–liver axis refers to a close anatomical, functional, and bidirectional interaction between the gastrointestinal tract, along with its microbiota, and the liver through the biliary tract, portal vein, and systemic circulation [87,88]. This axis appears to be a crucial functional component that protects the host against potentially dangerous and toxic chemicals from the intestine and maintains immune system homeostasis [89].

The liver communicates with the gut by releasing bile acids and antimicrobial molecules into the biliary tract and systemic circulation. In parallel, the metabolism of endogenous (bile acids, amino acids) and exogenous substrates (from diet and environmental exposure) by the host and microbiota results in the transportation of their products to the liver through the portal vein, affecting liver function [88,90]. The portal vein is the direct venous outflow from the gut that carries blood to the liver; from there, the liver redistributes and accumulates carbs, lipids, and amino acids as well as filtering the blood to remove gastrointestinal waste products [91].

Another important interaction between the gut and the liver is based on the metabolism of bile acids, which are synthesised in the liver from cholesterol and then released and reabsorbed in the gut by the microbiota [92]. The primary bile acids are then converted into secondary bile acids, facilitated by members of the GM, including the genera *Bacteroides*, *Clostridium*, *Eubacterium*, *Lactobacillus*, and *Escherichia* [93]. Approximately 95% of bile acids are reabsorbed at the distal ileum from the gut, transported back to the liver, and then discharged back into the intestine, generating a metabolic cycle known as enterohepatic circulation. Bile acids modulate the GM composition within this cycle and also have an impact on the liver’s metabolism and efficiency, indicating a two-way interaction between bile acids and the GM [93].

In a healthy state, an intact intestinal barrier prevents excessive bacterial translocation and restricts this direct host–microbiota contact [92]. On the other hand, when the gut barrier is compromised and has increased permeability, the liver is automatically exposed to a variety of hazardous substances coming from the gut as well as gut microorganisms, while these processes would be further enhanced by an intestinal dysbiosis [89]. Disruption of the gut–liver axis causes immune dysfunction that contributes to the development and progression of liver disorders [94].

### 3.1. Gut Dysbiosis in Liver Diseases

Disruption of the GM can affect the availability of SCFAs, gut permeability, bile acid metabolism, and glucose and lipid metabolism. It can also promote liver inflammation and injury. However, it is still unclear whether intestinal dysbiosis is a contributing factor to or a symptom of liver disorders [95]. GM dysbiosis has been associated with the progression of varying chronic liver diseases with distinct etiologies, including acute liver injury, viral hepatitis, non-alcoholic fatty liver disease (NAFLD), alcohol-related liver disease, autoimmune hepatitis (AIH), primary biliary cholangitis (PBC), and primary sclerosing cholangitis (PSC). The main dysbiotic events in the GM in representative liver diseases are outlined in Table 3.

#### 3.1.1. Dysbiosis in Non-Alcoholic Fatty Liver Disease (NAFLD)

NAFLD is the most frequent cause of chronic liver disease worldwide due to the rapidly increasing prevalence of obesity and metabolic syndrome. In NAFLD, in the absence of excessive alcohol consumption and other stimulating factors (e.g., drugs and viruses), the amount of fat accumulated in hepatocytes is greater than 5% of the weight of the liver [96]. NAFLD may progress to hepatic inflammation, resulting in non-alcoholic steatohepatitis (NASH), hepatic fibrosis/cirrhosis, and finally progressing to NAFLD-related hepatocellular carcinoma (NAFLD-HCC) [89]. Evidence from both preclinical and clinical studies suggests that GM dysbiosis plays a crucial role in the onset and maintenance of NAFLD.

The GM dysbiosis in NAFLD is characterised by a reduction in total bacterial diversity and richness and a disruption of the balance between Firmicutes and Bacteroidetes [95,97]. More specifically, an increase in the Bacteroidetes phylum, colonization by pro-inflammatory *Proteobacteria*, *Enterobacteriaceae*, and *Escherichia*, and a decrease in *Firmicutes* (including *Prevotella* and *Faecalibacterium* species) are the most common changes observed in NAFLD and NASH patients [95,98,99]. On the other hand, other studies found the opposite [100], highlighting the variability between studies, mainly resulting from the different demographic groups included in the studies as well as the different stages of NAFLD.

According to various studies, the types of GM dysbiosis in NAFLD patients vary by geographic region and gender [98,101]. In the study of Del Chierico et al. [101], where NAFLD patients and healthy subjects from Europe participated, NAFLD patients displayed increased abundance of *Bradyrhizobium*, *Anaerococcus*, *Peptoniphilus*, *Propionibacterium* acnes, *Dorea*, and *Ruminococcus*, while reduced abundance of *Oscillospira* and *Rikenellaceae* was observed compared to healthy subjects. In a cross-sectional study that examined the largest number of Asian patients, NAFLD patients had increased levels of Bacteroidetes and lower levels of Firmicutes than healthy controls [98]. Additionally, sex-specific differences in the GM in relation to NAFLD history have been observed [102]. Compared with controls, male NAFLD cases displayed reduced microbial α-diversity, increased abundance of genera *Dialister*, *Streptococcus*, and *Bifidobacterium* species, diminished abundance of the genus *Phascolarctobacterium*, and lower prevalence of taxa, including order RF39 and unclassified genus/species of families (*Mogibacteriaceae*), *Rikenellaceae*, and *Peptococcaceae*. On the other hand, female NAFLD cases displayed higher α-diversity, increased abundance of the genus *Butyricimonas* and a family of order Clostridiales, reduced abundance of *Dialister* and *Bifidobacterium* species, and an elevated prevalence of RF39 [102]. Furthermore, the relationship between gut dysbiosis and the severity of NAFLD lesions has also been studied by Boursier et al. [103]. The authors discovered that the severity of NAFLD is connected with gut dysbiosis, finding a higher abundance of *Bacteroides* in NASH patients compared to simple steatosis patients and a positive association between *Ruminococcus* abundance and severe fibrosis, independent of metabolic factors [103].

Overall, the above data suggest the involvement of the GM in NAFLD; however, it remains unclear whether GM dysbiosis is a direct cause of NAFLD or solely a reflection of disease-associated alterations in the host’s immune and metabolic systems.

#### 3.1.2. Dysbiosis in Cirrhosis

Liver cirrhosis is a common result of the protracted clinical course of all chronic liver diseases and is characterised by hepatocyte loss, fibrous scar thickening, and regenerative nodules [104]. GM dysbiosis has been linked to the etiology and manifestations of cirrhosis. Recent studies have shown differences in the GM between healthy subjects and patients with liver cirrhosis, and several groups have characterised the dysbiotic GM in cirrhosis, describing an obvious overrepresentation of pathogenic bacteria and fungi [105,106]. Chen et al. [107] conducted one of the first culture-independent surveys of the GM in cirrhotic patients. The authors found that the faecal microbial composition of patients with cirrhosis was distinct from that of controls, characterised by an increase in potentially pathogenic bacteria such as *Streptococcus*, *Veillonella*, and *Enterobacteriaceae* and a decrease in beneficial populations such as *Lachnospiraceae* [107]. A pivotal Chinese study observed ecological dysbiosis, where *Veillonella*, *Streptococcus*, *Clostridium*, and *Prevotella* were enriched in the cirrhosis group [108]. Among the 20 species that were enriched in the cirrhosis group, four were *Streptococcus* species and six were *Veillonella* species, including species originating from the oral cavity, indicating that the microbial source of liver cirrhosis is the translocation of oral bacteria to the intestine [108]. More recently, Sole et al. [109] used high-throughput analysis and found that marked alterations in the GM of cirrhotic patients, including a significant reduction of gene and metagenomic species richness and progressive enrichment by unusual gut bacteria, particularly *Enterecoccus* species, with some of them from the oral microbiota, were associated with the progression of cirrhosis with maximal changes in acute-on-chronic liver failure [109]. Even more recently, Huang et al. [110] found that cirrhotic patients had varying degrees of gut microbiome disorder, which was manifested by decreased *Lactobacillus* and *Bifidobacterium* counts and significantly increased *Enterobacter* and *Enterococcus* counts [110].

GM dysbiosis can be used as a prognostication tool for the diagnosis of liver cirrhosis [111]. In this context, the term “cirrhosis dysbiosis ratio (CDR)” was developed, which compares the ratio of beneficial bacteria (*Lachnospiraceae* + *Ruminococcaceae* + *Clostridiales Incertae Sedis XIV* + *Veillonellaceae*), to potentially pathogenic bacteria (*Enterobacteriaceae* + *Bacteroidaceae*), with a low number being indicative of dysbiosis and a high ratio indicating healthy microbiota [111]. In the same study, 244 subjects (219 cirrhotics (121 compensated outpatients, 54 decompensated outpatients, 44 inpatients) and 25 age-matched controls) were included. Controls had the highest CDR, followed by compensated patients, uncompensated patients, and inpatients. The GM was substantially different between infected and uninfected cirrhotic patients at baseline in the longitudinally matched cohort, and a low CDR was associated with death and organ failure within 30 days [111].

In addition, the faecal microbiota can be used as a prognostic tool for 90-day readmissions in cirrhosis. More specifically, in outpatients with cirrhosis, unique stool and salivary microbiome patterns have been associated with the risk of 90-day hospitalisation, regardless of the cause of hospitalisation [112]. Gut dysbiosis in cirrhosis may pathologically contribute to disease progression and decompensating events, such as spontaneous bacterial peritonitis (SBP) and hepatic encephalopathy (HE) [105]. Also, gut dysbiosis and its expansion to small intestinal bacterial overgrowth (SIBO) are observed in a significant proportion of patients with cirrhosis and is reported to be more prevalent in patients with advanced cirrhosis [113]. SIBO results in bacterial translocation, systemic inflammation, and hemodynamic changes that contribute to the development of cirrhosis complications such as ascites, hepatic encephalopathy (HE), oesophageal varices, and spontaneous bacterial peritonitis (SBP), indicating a dismal prognosis for SIBO patients [113,114].

Overall, GM dysbiosis in cirrhosis is associated with the onset and progression of liver disease, as well as specific clinical complications such as encephalopathy. Moreover, gastrointestinal microbial alterations are associated with increasing severity, susceptibility to infection, immune exhaustion, and hepatic and extrahepatic organ failures that result in acute decompensation or acute-on-chronic liver failure [115].

#### 3.1.3. Dysbiosis in Hepatocellular Carcinoma (HCC)

Hepatocellular carcinoma (HCC) is a frequent subtype of primary liver cancer, representing 75–85% of all primary liver cancers. HCC is a long-term consequence of chronic liver disease (CLD), and it develops primarily in cirrhotic livers, with the hepatitis B virus (HBV) or hepatitis C virus (HCV), diabetes, non-alcoholic fatty liver disease (NAFLD), alcoholism, as well as other genetic or metabolic disorders serving as its primary causes [116,117]. In addition, HCC develops in the dysbiotic and disrupted gut–liver axis characteristic of cirrhosis, which may change and deteriorate as HCC develops or advances. To date, the exact etiology and molecular mechanism of HCC have not been completely elucidated.

The relationship between the GM and the HCC has recently received increased attention, and existing studies suggest that the GM may be a candidate target for preventing and treating HCC [118]. GM dysbiosis has been studied in order to (1) characterise the GM in patients with HCC; (2) examine non-invasive biomarkers to diagnose primary HCC; and (3) prevent or treat primary HCC through the intestinal microbiota.

The GM has important potential as a non-invasive diagnostic biomarker to diagnose HCC. According to studies, Escherichia coli overgrowth may contribute to the formation of HCC, while the dysbiosis degree associated with primary HCC increases as the malignancy develops [116,119]. The study by Grat et al. [120] demonstrated that GM profiles related to HCC among cirrhotic patients were characterised by increased levels of *E. coli* in the faeces [120]. In the same line, Ni et al. [121] introduced an index referred to as the degree of dysbiosis (D_dys_), in order to identify GM alterations during the development of HCC. According to the results, patients with primary HCC had elevated levels of pro-inflammatory bacteria in their faecal microbiota and a significant increase in D_dys_ compared to healthy individuals [121]. In another study, 486 faecal samples were collected and subjected to 16S rRNA Miseq sequencing in order to evaluate the potential of the GM as a non-invasive biomarker for HCC [122]. A significant global shift in the GM from cirrhosis to HCC was observed, and the phylum Actinobacteria increased in early HCC versus cirrhosis. Also, 13 genera, including *Gemmiger* and *Parabacteroides*, were enriched in early HCC compared to cirrhotic patients, while 12 genera, including *Alistipes*, *Phascolarctobacterium*, and *Ruminococcus*, were reduced, and 6 genera were enriched in patients with early HCC compared to healthy controls. Finally, butyrate-producing genera, namely *Ruminococcus*, *Oscillibacter*, *Faecalibacterium*, *Clostridium IV*, and *Coprococcus*, were decreased, while genera producing lipopolysaccharides, *Klebsiella* and *Haemophilus*, were increased in early HCC versus controls [122].

Different biological pathways may be involved in HCC caused by different etiologies, as depicted by Liu et al. [123]. The authors conducted a cohort of 33 healthy controls, 35 individuals with HBV-related HCC (B-HCC), and 22 individuals with non-HBV non-HCV (NBNC)-related HCC (NBNC-HCC) through 16S rRNA analyses. According to the results, B-HCC patients displayed increased species richness compared to the other two groups. More specifically, B-HCC patients harboured significantly more pro-inflammatory bacteria, such as *Escherichia*/*Shigella* and *Enterococcus*, but less *Faecalibacterium*, *Ruminococcus*, and *Ruminoclostridium* than healthy controls and HBV-related HCC, suggesting that the gut–liver axis can be used to monitor and prevent the progression of liver disease and HCC [124].

### 3.2. The Role of Prebiotics in Liver Diseases

Prebiotics have been suggested as a treatment option for NAFLD, and several clinical trial results have demonstrated the therapeutic effects of modulating glucose homeostasis and lipid metabolism on the progression of NAFLD/NASH [125,126,127]. Additionally, the beneficial effects of prebiotics on NAFLD can be attributed to decreased de novo lipogenesis, weight and fat loss, enhanced blood glucose control, restored GM, and decreased inflammation [127]. The function of prebiotics in hepatocellular carcinoma has also been investigated [128,129]. The primary mechanisms include the production of SCFAs, which positively affect the structure and function of the intestinal microbiota, primarily by controlling colonocyte function, promoting water and electrolyte absorption, decreasing intraluminal pH, inhibiting pathogen proliferation, modifying the immune homeostasis of the gut, and modulating the inflammatory response [129]. In addition, the enhancement of intestinal barrier function by modifying GM composition ameliorates conditions such as cirrhosis and may therefore prevent HCC [128]. Despite the large number of studies examining the beneficial role of prebiotics in liver diseases, the administration of prebiotics to manipulate the GM as a new therapeutic approach has not been extensively investigated. Table 2 demonstrates the available GM manipulation-based interventions with prebiotics in liver diseases. The therapeutic potential of prebiotic supplements was examined in a placebo-controlled randomised pilot trial, where 14 individuals with liver-biopsy-confirmed NASH were randomised to receive oligofructose or an isocaloric placebo for 9 months [80]. Prebiotic supplementation improved liver steatosis relative to the placebo and improved the overall NAS score. Also, the prebiotic supplementation led to an increase in Bifidobacterium levels, while it resulted in a reduction of bacteria belonging to *Clostridium* cluster XI and I [80]. Benefits of lactitol to cirrhotic disease by profiling the GM and metabolites of cirrhotic patients before and after 4 weeks of lactitol treatment were demonstrated by Lu et al. [81]. According to the results, all clinical parameters, including MELD, showed no difference between pre- and post-lactitol treatment groups, while after the lactitol intervention, there was an increase in the levels of *Bifidobacterium longum*, *B. pseudocatenulatum*, and *Lactobacillus salivarius*. Additionally, there was a significant decrease in the pathogen Klebsiella pneumonia and the associated antibiotic-resistant genes and virulence factors [81].

Overall, there have been very limited data from clinical trials, and more human studies are needed in order to evaluate the role of prebiotics in NAFLD/NASH therapy, including for HCC prevention.

**Table 3:** Main dysbiotic events that occur in GM during the onset and progression of liver diseases.

Liver Disease	Main Dysbiotic Events in GM	Reference
Non-alcoholic fatty liver disease (NAFLD) and non-alcoholic steatohepatitis (NASH)	- The most common alterations observed in NAFLD and NASH patients include an increase in the *Bacteroidetes* phylum, colonisation by pro-inflammatory *Proteobacteria*, *Enterobacteriaceae*, and *Escherichia*, and a decrease in *Firmicutes* (including *Prevotella* and *Faecalibacterium* species) - The types of GM dysbiosis in NAFLD patients vary by geographic region and gender	[95,98,99,101]
Cirrhosis	- The dysbiotic GM in cirrhosis describes an overrepresentation of pathogenic bacteria and fungi such as *Streptococcus*, *Veillonella*, and *Enterobacteriaceae* and a decrease in beneficial populations such as *Lachnospiraceae* - GM dysbiosis can be used as a prognostication tool for the diagnosis of liver cirrhosis - Gut dysbiosis in cirrhosis may pathologically contribute to spontaneous bacterial peritonitis and hepatic encephalopathy - Gut dysbiosis and its expansion to small intestinal bacterial overgrowth (SIBO) are observed in patients with cirrhosis and are reported to be more prevalent in patients with advanced cirrhosis	[105,106,107,110,111,113]
Hepatocellular carcinoma (HCC)	- GM can be used as a non-invasive diagnostic biomarker to diagnose HCC, particularly with respect to the overgrowth of *Escherichia coli* that may contribute to the formation of HCC - The dysbiosis degree associated with primary HCC increases as the malignancy develops	[116,119,121,122]

## 4. The Gut–Lung Axis

For the longest time, the lung was thought to be a sterile organ, mainly because of limitations such as a lack of culture-independent approaches for microbial community profiling techniques and the risk of contamination from the oropharynx or nasal cavity through bronchoscopy techniques [130,131]. The advent of cutting-edge molecular methods for microbial characterization and metagenomic approaches revealed the detection of microbial DNA in the lungs of healthy individuals [132]. It is noteworthy that the upper and lower respiratory tract of healthy individuals differ significantly in the composition of the microbiota [133]. Additionally, the prevalence of distinct microbial species at these anatomical sites supports niche-specific microbial colonization at discrete anatomical sites [133,134]. However, the microbial community inhabiting the lungs is suggested to be partially seeded through microaspiration from the oral cavity. Budden et al. [131] support the hypothesis that entry and selective elimination of a transient microbiota rather than resident and viable microorganisms are the main determinants of microbial composition in the lungs.

The lungs are inhabited by a microbial population distinct from that of the gut [135]. Although the predominant bacterial phyla in the gut and lung microbiota are similar, mainly *Firmicutes* and *Bacteroidetes*, followed by *Proteobacteria* and *Actinobacteria* [136], they differ in their bacterial species composition [132]. The main bacterial genera found in the lungs include *Streptococcus*, *Prevotella*, and *Veillonella*, genera that are also found in the oral cavity [133].

The proposed pathways of the gut–lung interaction are not yet well established; however, they seem to involve the following mechanisms [137,138]: (1) Microbial cells and their products in the lamina propria that enter the intestinal mucosa are either subjected to phagocytosis and elimination or are transferred to mesenteric lymph nodes (MLNs) by antigen presenting cells (APCs), where they stimulate the activation of the T and B cells. (2) Activated B and T cells can migrate back to the intestinal mucosa to directly act on their target or to continue to trigger other immune cells, or via the lymphatic and blood circulation, they can move to distal sites such as the lung epithelium and lung nodes, to stimulate the immune system. Also, bacterial metabolites and the expression of antimicrobial peptides by epithelial cells fortify the intestinal barrier’s integrity. The same pathway has been proposed in the other sense, arising from lung mucosa with lung microbiota exerting effects on the gut; however, this mechanism is not yet well established in the literature.

### 4.1. Gut Dysbiosis in Pulmonary Diseases

Gut dysbiosis can lead to gut inflammation and increased gut permeability, allowing translocations of gut bacteria, bacterial components such as LPS, metabolites, pathogen-associated molecular patterns (PAMPs), cell wall components, and flagellin into the circulation and the lung, contributing to changes in lung immunity, including dysregulation of lung immune response by increasing inflammatory markers and T cell dysregulation. Dysbiosis contributes to an altered immune response, altered microbiota (a decrease in microbial diversity and pathogen multiplication), and tissue damage [139,140]. Microbial dysbiosis is associated with numerous lung diseases such as asthma, chronic obstructive pulmonary disease (COPD), lung cancer, and COVID-19; however, it is unclear whether gut dysbiosis is the cause of disease or a consequence of the disease process. Table 4 highlights the main dysbiotic events in GM composition identified in pulmonary disorders. Subsequent sections will delve into the analysis of representative diseases and their associated dysbiotic events.

#### 4.1.1. Dysbiosis in Asthma

Asthma is one of the most common chronic respiratory tract diseases, affecting people of all ages but usually beginning in childhood. Demographics, genetics, and environmental factors have been identified as the main risk factors for asthma development [141]. Asthma has multiple phenotypes, with different pathophysiological and clinical characteristics, including wheezing, coughing, shortness of breath, chest tightness, and expiratory airway limitation, and an even wider variety of underlying molecular and immunological mechanisms, known as endotypes [130,141]. The two asthma endotypes that are best described are the type 2 endotype, which consists primarily of Th2 cell responses, and the non–type 2 endotype [130]. Type 2 asthma typically manifests as early-onset allergic asthma, late-onset eosinophilic asthma, or exercise-induced asthma, whereas non–type 2 asthma mechanisms typically manifest as neutrophilic, obesity-related, and paucigranulocytic phenotypes [130]. The prevalence of asthma remains high, affecting more than 300 million individuals worldwide and is expected to increase to 400 million by 2025 [142].

Numerous studies have demonstrated that microbial dysbiosis and diminished microbial diversity have been identified as triggers of gut–lung axis dysregulation and linked to the development of asthma [143,144,145]. Growing evidence suggests that a deficiency in gut microbial composition in early life is associated with childhood asthma development, as the GM has a significant influence on immune cell maturation and resistance to pathogens [141]. The first systematic review to evaluate the association between early-life GM and childhood respiratory diseases, including asthma, was conducted by Alcazar et al. [146]. The main finding was that a low relative abundance of *Bifidobacterium* in faeces collected before the age of 3 months was associated with asthma in children aged 4–5 years. In addition, a low abundance of the genera *Faecalibacterium*, *Roseburia*, and *Ruminococcus* in stool samples collected between the ages of 3 and 12 months was associated with asthma between the ages of 1 and 6 years.

Numerous research groups have demonstrated alterations in the GM of healthy subjects compared to those of asthma patients in terms of composition and diversity [147,148,149]. In the study of Zou et al. [149] faecal samples were collected from 20 healthy subjects and 47 newly diagnosed asthmatic patients. The results demonstrated that asthmatic patients have a higher abundance of *Ruminococcus gnavus*, *Bacteroides plebeius*, and *Clostridium clostridioforme* and a lower abundance of *Roseburia inulinivorans* and *Clostridium disporicum*. Also, differences were observed between allergic and non-allergic subjects. More recently, in another study, faecal samples derived from 13 asthma patients and 7 healthy volunteers were analysed using Next-Generation Sequencing technology. The results showed significant differences in the human gut microbiome composition between asthma patients and the healthy control group at the genus and species level. Patients with asthma showed a significantly greater population of *Parabacteroides*, *Paenibacillus*, *Sulfurimonas*, and *Lachnoclostridium*, while the healthy group had a greater population of *Faecalitalea*, *Haemophilus*, *Syntrophothermus*, *Methanocella*, and *Geobacter* [147].

Moreover, studies have shown that alterations in gut microbial composition are involved in the severity of asthma [150]. A lower abundance of *Acidaminococcaceae* and a higher abundance of *Veillonellaceae* and *Prevotellaceae* was observed in severe asthma, while the abundance of *Veillonellaceae* was related to lung function [150]. Although the mechanisms mediating communication between the gut and lungs remain unclear, it has been hypothesised that epithelial cells, other structural cells, and immune cells absorb signals from the gut endothelium to form a local cytokine microenvironment, resulting in changes in immune responses at distal sites [151]. Also, SCFAs derived from the GM specifically inhibit proinflammatory lung responses.

#### 4.1.2. Dysbiosis in Chronic Obstructive Pulmonary Disease (COPD)

Chronic Obstructive Pulmonary Disease (COPD) is a progressive, chronic lung disease that affects over 400 million people globally and is responsible for 3 million deaths each year [152]. COPD is characterised by increasing shortness of breath, chronic cough, and sputum production, which are accompanied by an irreversible progressive inflammatory condition and significant lung tissue damage (emphysema) with airflow obstruction [153]. The classification of severity of COPD includes four stages based on spirometry and the degree of worsening airflow limitation from stages I to IV. The mechanism of pathogenesis remains unknown; however, various environmental factors are involved, including chronic aeropollutant exposure, primarily from cigarette smoking, and bacteria or virus infection [154].

Although limited, growing evidence suggests that dysbiosis of the GM is a crucial factor in the pathophysiology of COPD [139,155]. Recent studies have shown that the faecal microbiota derived from COPD patients differs from that of healthy subjects. In the study of Li et al., 2021 [155], the authors performed 16S ribosomal RNA sequencing of faecal samples from COPD patients and healthy controls. According to the results, the relative proportion of Bacteroidetes was lower and that of Firmicutes was higher in the COPD group when compared to healthy subjects, while at the family level, they demonstrated differing relative abundances between groups for *Fusobacteriaceae*, *Prevotellaceae*, and *Bacteroidaceae*, whereas *Prevotella* were enriched in the faeces of COPD patients [155]. In the study of Bowerman et al. [156], several *strepotococci* species, including *Streptococcus* sp000187445, *Streptococcus vestibularis*, and members of the family *Lachnospiraceae*, distinguish between COPD patients and healthy controls and are also correlated with impaired lung function [156]. Also, GM alterations between different stages of COPD have been observed [157]. More particularly, the authors reported that among patients with COPD, patients with rapid lung function decline had an increased abundance of Firmicutes and a declining abundance of *Bacteroidetes* and *Alloprevotella*. In addition to a decline in lung function, the authors demonstrated that the mean proportions of *Acinetobacter* and *Stenotrophomonas* significantly increased.

Also, the GM may be involved in the acute exacerbations of COPD, resulting in negative effects [136,154]. Sprooten et al. [158] demonstrated that patients with hospitalised acute exacerbations of COPD have disrupted intestinal permeability compared to patients with stable COPD after a 4-week recovery period. Furthermore, Otigger et al. [159] demonstrated an association between increased circulating trimethylamine N-oxide (TMAO) levels and long-term all-cause mortality in COPD patients, independent of the type of exacerbation. According to the above, the GM may play a vital role in the pathogenesis of COPD. However, additional research is required to determine the exact relationships between gut microbiome diversity and COPD pathophysiology.

#### 4.1.3. Dysbiosis in Severe Acute Respiratory Syndrome Coronavirus 2 (SARS-CoV-2)

COVID-19 (coronavirus disease 2019), related to the severe acute respiratory syndrome coronavirus 2 (SARS-CoV-2), is caused by a novel RNA virus of the family Coronaviridae. In December 2019, Wuhan, China, recorded the first-ever case of the COVID-19 outbreak, leading to a global pandemic within four months [160]. As of 30 August 2023, there had been 770,085,713 confirmed cases of COVID-19 across the world reported to the World Health Organization (WHO), including 6,956,173 deaths [161]. Clinical presentations of COVID-19 vary greatly, ranging from no or mild symptoms, mild respiratory tract illness, and severe pneumonia to more severe cases, including respiratory, hepatic, gastrointestinal, and neurological complications that require hospitalization and can eventually progress to multi-organ dysfunction and death [162]. The most typical respiratory symptoms are fever, dry cough, fatigue, and dyspnoea [162]. In addition to respiratory symptoms, gastrointestinal symptoms, such as diarrhoea, nausea, and vomiting, are prevalent in COVID-19 patients, with some patients reporting only gastrointestinal symptoms [163].

A relationship between the GM and COVID-19 has been established by multiple studies. The primary findings indicate that GM dysbiosis either is involved in the disease’s development or progression or occurs due to COVID-19 [164]. The main findings that highlight the involvement of intestinal microbiota dysbiosis in COVID-19 include the following: 

(1) The dysbiotic GM composition of COVID-19 patients [160,165,166]. In a study by Gaibani et al. [167], the GM of COVID-19 patients appeared seriously dysbiotic, enriched in potential pathogens (e.g., *Actinomyces*, *Akkermansia*, *Collinsella*, *Enterococcus*, *Lactobacillus*, *Lactococcus*, *Methanobrevibacter*, *Odoribacter*, *Parabacteroides*, *Phascolarctobacterium*, *Serratia*, and *Staphylococcus*), with reduced diversity and loss of beneficial microorganisms, mainly *Bacteroides*, *Blautia*, *Coprococcus*, *Dialister*, *Faecalibacterium*, *Lachnospira*, *Oscillospira*, *Prevotella*, *Roseburia*, and *Ruminococcus*. In a systematic review, they found that the GM diversity of COVID-19 patients in both the acute and recovery phases was consistently lower than that of non-COVID-19 individuals. More specifically, a decrease in anti-inflammatory butyrate-producing bacteria (*Megasphaera*, *Dialister*, *Ruminococcus*, *Faecalibacterium*, *Roseburia*, *Lachnospira*, and *Prevotella*) was observed in COVID-19 patients [168].

(2) The dysbiosis of gut microbiome profile of COVID-19 patients has been found to be correlated with disease severity. Lymberopoulos et al. [169] demonstrated an association between anti-inflammatory bacteria, such as *Bifidobacteria* species and *Eubacterium rectale*, and decreased COVID-19 severity, and between pro-inflammatory bacteria, such as *Prevotella copri*, and increased COVID-19 severity, highlighting COVID-19 severity associations with population-level gut microbiome variations.

(3) The detection of SARS-CoV-2 in COVID-19 patients’ faeces. Faecal collection derived from patients with COVID-19 showed depletion of symbionts and enrichment of opportunistic pathogens, *Collinsella aerofaciens*, *Collinsella tanakaei*, *Morganella morganii*, and *Streptococcus infantis*, which persisted even after clearance of SARS-CoV-2 and resolution of respiratory symptoms [170]. (4) Gut dysbiosis persists even after recovery from COVID-19 and may contribute to long COVID-19. Chen et al. [171] demonstrated that after six months of recovery, microbiota richness had not returned to normal levels. Patients with attenuated postconvalescence richness exhibited higher levels of C-reactive protein (CRP) and illness severity during the acute phase, indicating a strong correlation between inflammatory response and gut dysbiosis in COVID-19. In a recent study by Zhang et al. [172], the presence of long COVID-19 correlates with GM dysbiosis, including significantly diminished bacterial diversities and a lower relative abundance of SCFAs-producing salutary symbionts such as *Eubacterium_hallii*_group, *Subdoligranulum*, *Ruminococcus*, *Dorea*, *Coprococcus*, and *Eubacterium_ventriosum*_group in recovered patients at one year after discharge.

To document the relationship between the human gastrointestinal microbiota and the clinical effects of acute infection, which may persist even after viral RNA clearance, additional comparative and longitudinal studies of larger cohorts are required.

#### 4.1.4. Dysbiosis in Lung Cancer

Lung cancer is one of the most prevalent malignant tumours and was the leading cause of cancer-related death in 2020, accounting for approximately 18% of all cancer-related deaths [173]. Non-small cell lung cancer (NSCLC) and small cell lung cancer (SCLC) are the two most common histological-pathological subtypes. Surgery, chemotherapy, radiotherapy, and emerging immunotherapies are used to treat lung cancer [174]. Genetic and environmental factors are the major etiological causes facilitating the pathogenesis of lung cancer, whereas the key determinant remains tobacco consumption [173]. Among diverse environmental risk factors, researchers have recently focused on the GM for a novel lung cancer prevention and treatment breakthrough [175].

Although few studies have been conducted on the GM characteristics of lung cancer patients, similar acknowledgments have been made. More specifically, the main findings of the studies include the following [123,175,176]: 

(1) Significant alterations in GM composition and function in lung cancer patients. Zhuang et al. [177] stated that increased levels of Enterococcus in the GM are associated with lung cancer, while Bifidobacterium and Enterococcus were found to be the highest potential biomarkers for lung carcinogenesis. Liou et al. [123] analysed the faecal microbiota collected from 16 healthy individuals and 30 lung cancer patients, who were divided into three groups based on different tumour biomarkers. According to the results, the gut microbial community of each lung cancer group exhibited low abundance and low bacterial diversity compared to that of the healthy group, characterised by a diverse and special pathogen microbiome such as *Enterobacteriaceae*, Streptococcus, *Prevotella*, etc., and fewer beneficial genera, including *Blautia*, *Coprococcus*, *Bifidobacterium*, and *Lachnospiraceae*. In another study, lung cancer patients had lower abundances of *Firmicutes* and *Proteobacteria*, along with relatively higher levels of *Bacteroidetes* and *Fusobacteria*, compared to healthy subjects [176].

(2) GM dysbiosis may impact lung cancer treatment and prognosis. Routy et al. [178] conducted metagenomic analyses of lung cancer patient stool samples and reported positive correlations between immune checkpoint inhibitors (ICIs) targeting the PD-1/PD-L1 axis and the relative abundance of *Akkermansia muciniphila*. In addition, oral supplementation with *Akkermansia muciniphila* increased the response to immune checkpoint inhibitors, whereas a dysbiotic GM was implicated in resistance to ICI treatment [178]. A recent study by Tomita et al. [179] revealed that probiotic consumption of *Clostridium butyricum* MIYAIRI 588 strain significantly improved progression-free survival and overall survival in patients with non-small cell lung cancer treated with immune checkpoint blockade (ICB), compared to those not treated with probiotic CBT, suggesting that manipulating the GM by probiotic CBT has the potential to enhance the efficacy of ICB [179].

Taken together, the above studies indicate the role of the GM as a potential biomarker for the diagnosis and treatment of lung cancer. However, more studies are needed to explore the role of the GM in the development and progression of lung cancer.

### 4.2. The Role of Prebiotics in Lung Diseases

Several studies highlight the effectiveness of the use of prebiotics in viral diseases, including respiratory diseases; however, the majority of these studies involve individuals in infancy [180,181]. Also, the above studies do not record the effect of prebiotics on the GM of the volunteers. To date, there are no studies that demonstrate GM manipulation-based interventions with prebiotics, especially on asthma, COVID-19, COPD, and lung cancer, though the potential of prebiotics for the prevention and treatment of the above diseases has been demonstrated in review papers [182,183,184]. Therapeutic approaches targeting the GM are required to examine the potential preventive or/and curative effects of prebiotics against pulmonary diseases.

**Table 4:** Main dysbiotic events that occur in GM during the onset and progression of pulmonary diseases.

Pulmonary Disease	Main Dysbiotic Events in GM	Reference
Asthma	- Deficiency in GM composition in early life is associated with childhood asthma development - Asthmatic patients have a higher abundance of *Ruminococcus gnavus*, *Bacteroides plebeius*, and *Clostridium clostridioforme* and a lower abundance of *Roseburia inulinivorans* and *Clostridium disporicum* - Alterations in GM composition are involved in the severity of asthma; mainly, a lower abundance of *Acidaminococcaceae* and a higher abundance of *Veillonellaceae* and *Prevotellaceae* was observed in severe asthma	[141,146,147,148,149]
Chronic obstructive pulmonary disease (COPD)	- In individuals with COPD compared to healthy subjects, there was a decrease in the proportion of *Bacteroidetes* and an increase in *Firmicutes*. Additionally, at the family level, there were notable differences in the relative abundances of *Fusobacteriaceae*, *Prevotellaceae*, and *Bacteroidaceae,* whereas *Prevotella* was more prevalent in the faecal samples of COPD patients - Specific *Streptococci* species and members of the *Lachnospiraceae* family were identified as distinguishing factors between COPD patients and healthy controls, and were also found to be correlated with impaired lung function - GM may be involved in the acute exacerbations of COPD, resulting in negative effects	[136,154,155,156]
Severe acute respiratory syndrome coronavirus 2 (SARS-CoV-2)	- The GM of COVID-19 patients appeared seriously dysbiotic, enriched in potential pathogens, with reduced diversity and loss of beneficial microorganisms, mainly *Bacteroides*, *Blautia*, *Coprococcus*, *Dialister*, *Faecalibacterium*, *Lachnospira*, *Oscillospira*, *Prevotella*, *Roseburia*, and *Ruminococcus* - GM dysbiosis of COVID-19 patients has been found to be correlated with disease severity - Gut dysbiosis persists even after recovery from COVID-19 and may contribute to long COVID-19	[167,169,171,172]
Lung cancer	- Augmented levels of *Enterococcus* in GM are associated with lung cancer, while *Bifidobacterium* and *Enterococcus* were found to be the highest potential biomarkers for lung carcinogens - GM dysbiosis may impact lung cancer treatment and prognosis	[177,178,179]

## 5. The Gut–Heart Axis

In recent years, the interaction between the GM and the heart has garnered considerable attention, as mounting evidence has demonstrated that the GM plays an essential role in cardiovascular diseases [185,186]. The GM is involved in a complex, bidirectional cross-talk with the heart, and the interaction occurs via bacterial metabolites that are produced from food components, which are resorbed in the gut and distributed in the circulation. Some of these metabolites, such as TMAO, can exacerbate cardiovascular pathologies [187].

In particular, within this axis, various microorganisms modulate metabolic reactions by producing bile acids, choline, and short-chain fatty acids, which are essential to host health. These metabolites are significant because they influence the metabolic phenotype of the host and the disease’s development risk. In addition, dietary or environmental changes may have an impact on health risk or disease by causing alterations in the composition or diversity of the GM [188]. Consequently, the signals transmitted by microorganisms and their components or agents induced and secreted by intestinal epithelial cells (IECs) or intestinal dendritic cells play an essential role in the physiological and pathophysiological functions of the host [10]. The gut–heart connection has recently been proposed to represent one of the newest targets for the prevention and treatment of cardiovascular disorders, including hypertension, atherosclerosis, cardiomyopathy, and heart failure [189].

### 5.1. Gut Dysbiosis in the Gut–Heart Axis

Dysbiosis of the GM may result in decreased cardiac function and increased cardiomyopathy, as well as cardiac insufficiency, which is highly predictive of cardiovascular diseases (CVDs) and adverse cardiovascular events [190]. In the development of cardiovascular pathogenesis due to accelerated dysbiosis, many critical factors sequentially play a role, including the GM, intestinal permeability, gut-derived substances (e.g., metabolites, toxins, and peptides), the immune system, and cellular components of the cardiovascular system [191].

Across the intestinal barrier, GM imbalance contributes to altered host metabolites and cytokine production, which in turn stress the cellular components of the heart and vasculature, thereby increasing cardiovascular risks [191]. In addition to the gut–heart axis, the gut microbiome may disrupt cardiovascular homeostasis via the gut–liver and gut–brain axis [192]. Also, the GM is associated with cardiovascular disease risk factors such as obesity, type 2 diabetes, and insulin resistance. These risk factors may influence the composition and diversity of the GM, while GM dysbiosis is also linked to inflammation, oxidative stress, platelet activity, thrombosis, and atherosclerosis, all of which contribute to the development of cardiovascular disease [190]. The possible mechanism of GM dysbiosis associated with cardiovascular diseases involves the participation of intestinal microbiota in increasing intestinal permeability and inducing inflammation via the LPS/TLR4 signalling pathways and the NLRP3 pathways, thereby contributing to the development of cardiovascular diseases [193]. Table 5 provides a summary of the main dysbiotic events in the GM identified in representative heart diseases.

#### 5.1.1. Gut Dysbiosis in Hypertension

Hypertension is the most prevalent modifiable risk factor for worldwide morbidity and mortality and a significant risk factor for cardiovascular disease [191]. The pathogenesis of hypertension is complex and influenced by environmental and genetic factors; while excessive salt intake is associated with elevated blood pressure (BP), a low-sodium diet reduces BP and morbidity and mortality from CVD [194]. The most notable features of the GM in hypertensive patients are the reduction in microbial diversity and richness, altered microbial structure and function, compositional change of taxa, and alterations in nutritional and immunological factors as well as microbial interactions [190,195,196]. In patients with hypertension, beneficial bacteria such as *Faecalibacterium*, *Bacteroides*, *Roseburia*, *Bifidobacterium*, *Coprococcus*, and *Butyrivibrio* were reduced, whereas *Veillonella*, *Prevotella*, and *Klebsiella* were elevated, according to several studies [197,198,199].

Interestingly, decreased diversity of the GM was found in hypertension, pre-hypertension, preeclampsia, and pulmonary arterial hypertension patients [195]. A potential novel causal role of a dysbiotic GM in contributing to the pathogenesis of hypertension was demonstrated by Li et al. [198]. The authors discovered significantly reduced microbial richness and diversity, with the *Prevotella* strain dominating the gut enterotype, distinct metagenomic composition with reduced health-related bacteria (*Faecalibacterium*, *Oscillibacter*, *Roseburia*, *Bifidobacterium*, *Coprococcus*, and *Butyrivibrio*), and overgrowth of bacteria such as *Prevotella* and *Klebsiella*, in both pre-hypertensive and hypertensive populations compared to healthy controls. In addition, microbiome analysis revealed that the activity of genes involved in amino acid synthesis, fatty acid utilisation, and saccharide transport decreased, while the biosynthesis of such metabolites increased [198].

An additional crucial evaluation for microbiota dysbiosis was the examination of differential microbial groups by comparing the hypertension group with the healthy control group. Also, in hypertensive patients, the alpha diversity of the microbiota and the abundance of short-chain fatty-acid-producing microbiota have been found to be diminished [197,200,201]. Kim et al. [202] analysed 40 faecal samples from 22 hypertensive individuals and 18 normal controls and discovered that *Parabacteroides johnsonii*, *Eubacterium siraeum*, and *Alistipes finegoldii* were present at a higher abundance in patients with hypertension, whereas butyrate-producing *Bacteroides thetaiotaomicron* was present at a lower abundance in the hypertensive group. In a recent study, Palmu et al. [203] demonstrated that 45 microbial genera were positively associated with blood pressure indexes, including 27 belonging to the Firmicutes, while they reported significant negative associations between 19 different Lactobacillus species and blood pressure indexes.

In recent years, a lot of research data have indicated that targeting the intestinal microbiota for the treatment of hypertension with pre/pre/symbiotics, lifestyle modifications, and diet is effective [204]. However, additional research is required to better understand the function of various gut microbial species and their metabolites in blood pressure regulation and related diseases.

#### 5.1.2. Gut Dysbiosis in Atherosclerosis

Atherosclerosis (AS), the pathological basis of various CVDs, is a chronic inflammatory disease and remains the leading cause of death worldwide. The pathogenesis of atherosclerosis is characterised by the accumulation of fatty and fibrous material in the intimal layer of arteries, which leads to the proliferation of fibres and calcium deposition, progressively thickening and hardening the walls of blood vessels [190]. Many risk factors for AS, such as lifestyle, dietary patterns, ageing, and obesity, share dysbiosis of the gut as a common denominator [205]. The following are some of the mechanisms by which the GM may affect the onset of atherosclerosis [206]: (1) the production of compounds resulting from the interaction of the GM and nutrition, which may alter the inflammatory and immune response that affects the atherosclerotic process; (2) the production of SCFAs; (3) the regulation of lipid metabolism, which influences the development of atherosclerosis through bile acid metabolism; (4) the preservation of intestinal barrier integrity, which prevents the absorption of pro-inflammatory bacterial components like LPS; and (5) the alteration of the functional metabolism of important hormones.

The presence of bacterial DNA in atherosclerotic plaques in human endarterectomy specimens shed the first light on gut microbiota’s involvement [207,208]. *Chryseomonas* was identified in all atherosclerotic plaque samples, along with *Veillonella* and *Streptococcus* in the majority of them, according to the 2011 study by Koren et al. [207]. Several bacterial species were common in the atherosclerotic plaque and oral or gut samples from the same individual, and several oral and gut bacterial taxa were correlated with plasma cholesterol levels. The observation that DNA from several bacterial species is present in both atherosclerotic lesions and the GM of the same individuals suggests that the GM may be a potential source of atherosclerotic bacteria and therefore likely plays a role in the pathogenesis of coronary artery disease [207].

In addition, researchers have found that the intestinal microbiota of patients with atherosclerosis differs from that of individuals without atherosclerosis. In the study by Jie et al. [209], stool samples from 218 individuals with atherosclerotic cardiovascular disease (ACVD) and 187 healthy controls were analysed. The results demonstrated that the GM of ACVD patients deviated from its healthy status due to an increased abundance of Enterobacteriaceae and *Streptococcus* spp. Furthermore, metagenomic analyses demonstrated elevated relative abundance of the genus *Collinsella* in the faecal samples of patients with symptomatic atherosclerosis, whereas butyrate-producing bacteria *Roseburia* and *Eubacterium* were enriched in healthy controls, indicating a dysbiosis condition in atherosclerotic patients [210].

The above findings highlight the role of certain gut bacteria as new factors that contribute to the progression of atherosclerosis, while other bacteria can act protectively against atherosclerotic plaque lesions. It is still unknown how these microorganisms can lead to or initiate the development of atherosclerosis, and the detailed mechanisms involved require further investigation.

#### 5.1.3. Gut Dysbiosis in Heart Failure (HF)

Heart failure (HF), which encompasses a group of complex clinical syndromes, is a severe and terminal stage of many cardiovascular diseases that result in damage to the structure or function of the heart [211,212]. In recent years, several studies have demonstrated the role of intestinal microbiota in the pathogenesis of heart failure disease, which is often referred to as the “gut hypothesis of heart failure” [211,213]. The gut hypothesis implies that decreased cardiac output and redistribution of systemic circulation can lead to a disrupted intestinal mucosa, which in turn can lead to increased gut permeability, increased bacterial translocation, and increased circulating endotoxins, which can contribute to the underlying inflammation seen in patients with HF [211,213].

Numerous studies have attempted to characterise the GM profile in heart failure, and the main findings include (1) significant alterations consistent with bacterial overgrowth; (2) a shift towards pathogenic phyla; and (3) a decrease in anti-inflammatory bacteria [214]. Different studies reported significant differences between the gut microbial composition of heart failure patients and healthy controls. Pasini et al. [215] compared the bacteria and fungi (*Candida* species) in the faeces of HF patients with those of healthy controls. The results showed that patients with chronic heart failure were colonised by more pathogenic bacteria (*Campylobacter*, *Shigella*, *Salmonella*, and *Yersinia*) than the control patients. In another study, heart failure cases showed a significant decrease in *Coriobacteriaceae*, *Erysipelotrichaceae,* and *Ruminococcaceae* on the family level and a significant decrease in *Blautia*, *Collinsella*, *unclassified (uncl.) Erysipelotrichaceae*, and *uncl. Ruminococcaceae* at the genus level [216]. Kamo et al. [217] analysed the GM of HF patients and healthy control subjects using 16S ribosomal RNA gene sequencing. The authors demonstrated a reduction in SCFA-producing bacteria, such as *Eubacterium rectale* and *Dorea longicatena*. Moreover, the research highlighted that the GM composition of older HF patients differed from that of younger HF patients.

Furthermore, gut diversity decreases across worsening HF class, as demonstrated by the study of Yuzefpolskaya et al. [218]. The authors evaluated the GM of HF patients with different degrees of severity and demonstrated that alpha diversity was reduced as disease severity levels increased. Similarly, in a recent study by Zhang et al. [219], the GM was altered with different grades of chronic heart failure. In addition, the depletion of SCFA-producing bacteria in HF patients was demonstrated by several studies. The majority of the depleted microorganisms in HF belonged to the *Lachnospiraceae* family, in addition to *Faecalibacterium* from the *Ruminococcaceae* family, indicating a diminished capacity for butyrate production [220]. Further large-scale longitudinal studies to identify a relationship between gut dysbiosis and heart failure must be conducted.

### 5.2. The Role of Prebiotics in Heart Diseases

Numerous studies have examined the potential positive benefits of prebiotics on host metabolism to ameliorate cardiovascular disease states, emphasising three potential mechanisms: (1) lowering blood lipids; (2) reducing endotoxemia and inflammation; and (3) lowering blood pressure [221,222,223,224]. However, the effect of prebiotics on the prevention and treatment of cardiovascular disease is often based on brief reports and small-scale clinical studies, and the mechanisms have not been clearly elucidated. The majority of the above studies do not examine the effects and possible changes in the GM. To our knowledge, only one study has examined the effects of prebiotics on individuals with hypertension and their ability to lower blood pressure in patients with essential hypertension and gut microbiome modulation [82] (Table 2). More specifically, in the study of Jama et al. [82], the results of a phase II, randomised, placebo-controlled, double-blind cross-over trial using prebiotic acetylated and butyrylated high-amylose maize starch (HAMSAB) supplementation in untreated patients with hypertension were presented. A 3-week intervention with HAMSAB resulted in a reduction in ambulatory systolic blood pressure and also promoted the growth of the commensal bacteria *P. distasonis* and *R. gauvreauii* and supported the restoration of local production of SCFAs by these microbes. The potential underlying mechanisms by which prebiotics lower the risk of hypertension include reducing lipid and cholesterol synthesis by increasing SCFA production; reducing obesity through improving satiety and lowering food intake through the gut’s production of endogenous glucagon-like peptide-1; enhancing hepatic insulin sensitivity and reducing insulin resistance by SCFAs; and improving the digestive tract’s ability to absorb nutrients [225]. The effect of prebiotics as a dietary strategy to modulate GM synthesis to protect against atherosclerosis and heart failure has not been examined. It is considered necessary to carry out clinical studies on a large scale in order to investigate the possible beneficial effects of the administration of prebiotics in the above diseases.

**Table 5:** Main dysbiotic events that occur in GM during the onset and progression of heart diseases.

Heart Disease	Main Dysbiotic Events	Reference
Hypertension	- In individuals with hypertension, there was a reduction in beneficial bacteria, including *Faecalibacterium*, *Bacteroides*, *Roseburia*, *Bifidobacterium*, *Coprococcus*, and *Butyrivibrio*, whereas elevated levels of *Veillonella*, *Prevotella*, and *Klebsiella* were observed - In patients with hypertension, there was an increased abundance of *Parabacteroides johnsonii*, *Eubacterium siraeum*, and *Alistipes finegoldii*, whereas butyrate-producing *Bacteroides thetaiotaomicron* was notably reduced in the hypertensive group	[198,199,202,204]
Atherosclerosis (AS)	- GM may be a potential source of atherosclerotic bacteria and therefore likely plays a role in the pathogenesis of coronary artery disease - The GM of atherosclerotic cardiovascular disease patients displayed an increased abundance of *Enterobacteriaceae* and *Streptococcus* spp. - In patients with symptomatic atherosclerosis, there was an increased relative abundance of the genus *Collinsella* in their faecal samples	[207,209,210]
Heart failure (HF)	- Patients with chronic HF were colonised by more pathogenic bacteria, including *Campylobacter*, *Shigella*, *Salmonella*, and *Yersinia,* and there was a reduction in SCFA-producing bacteria such as *Eubacterium rectale* and *Dorea longicatena* compared to healthy controls	

## 6. The Gut–Kidney Axis and Dysbiosis

The gut–kidney axis represents a crucial interplay between the gastrointestinal tract and the kidneys and is mediated through metabolism-dependent and immune pathways [226]. Through the symbiotic relationship, the intestine facilitates the absorption of beneficial microbial metabolites, while the kidneys play a role in maintaining equilibrium by excreting potentially toxic metabolic end-products. Conversely, following dietary exposure to specific nutrients, the host’s GM can elicit both metabolism-dependent and immune pathways. In the metabolism-dependent pathway, an unbalanced diet causes dysbiosis, which results in the overproduction and accumulation of p-cresyl and indoxyl sulphates in the gut [226,227]. Consequently, it disrupts the gut barrier, thus increasing permeability. As a result, endotoxins and uraemic toxins enter the kidneys through circulation, leading to inflammation in the renal system [227]. The immune-dependent pathway includes immune cells originating from the bone marrow that encounter dysbiotic microbiota and thus trigger an immune response, leading to the activation of various immune cells [226,228]. As a result, inflammation-induced molecules, including inflammatory cells, cytokines, and the soluble urokinase plasminogen activator surface receptor (suPAR), are transported through the bloodstream and contribute to renal inflammation [226,228]. Gut-derived uremic toxins, including p-cresyl and indoxyl sulphates, and TMAO, have been associated with the progression of CKD and an increased cardiovascular risk [229]. The pathogenic interplay between the GM and kidney seems to be involved in a wide range of clinical manifestations, such as chronic kidney disease (CKD), acute kidney injury (AKI), hypertension, kidney stone disease, nephropathy, haemodialysis, and peritoneal dialysis. Table 6 provides a summary of the main dysbiotic events in the GM identified in representative kidney diseases.

### 6.1. Gut Dysbiosis in Chronic Kidney Disease (CKD)

Chronic kidney disease (CKD) is the most widespread kidney disease. It affects around 10% of the world’s population and causes a significant burden on society, the economy, and the healthcare system [230]. The progressive loss of kidney function that characterises CKD causes changes in the blood levels of various toxic substances that are typically metabolised and excreted by the kidney. As a result, those substances, as well as elevated urea concentrations, consequently accumulate in the blood and cause uraemia [231]. CKD frequently co-occurs with other comorbidities, and if left untreated, it can progress to end-stage kidney disease (ESKD). Currently, there is no effective treatment, and the options for managing CKD in ESKD are limited to dialysis and kidney transplantation [228].

Many studies have examined and highlighted the key role of the GM as a mediator in the onset of CKD, and the main findings of the studies include the following: 

(1) Quantitatively and qualitatively, GM alterations such as a decrease in microbial richness, diversity, and uniformity occurred in CKD patients [232]. Patients with CDK display increased intestinal levels of *Lachnospiraceae*, *Enterobacteriaceae* (particularly *Enterobacter*, *Klebsiella*, and *Escherichia*), *Enterococci*, *Clostridium perfringes*, and certain *Ruminococcaceae*, and decreased levels of *Prevotellaceae*, *Bacteroidaceae*, and particularly *Lactobacillus* and *Bifdobacterium* species [233,234]. The most consistently observed changes in the GM of CKD patients involve reduced levels of *Lactobacillaceae* and *Bifidobacteriaceae*, along with elevated levels of *Enterobacteriaceae* [232]. In the study conducted by Vaziri et al. [235], faecal DNA was isolated from 24 patients with end-stage renal disease (ESRD) on haemodialysis and 12 healthy individuals and analysed using phylogenetic microarray techniques. The authors found that uraemia profoundly alters the GM composition and observed significant differences in the frequency of these samples between ESRD patients and healthy controls. Specifically, operational taxonomic units (OTUs) belonging to *Brachybacterium*, *Catenibacterium*, *Enterobacteriaceae*, *Oraxellaceae*, *Halomonadaceae*, *Pseudomonadaceae*, *Nesterenkonia*, *Polyangiaceae*, and *Thiothrix* were notably elevated in ESRD patients. Conversely, the families *Prevotellaceae* and *Lactobacillaceae* showed significant decreases. The characteristics of the GM in CKD patients who are not undergoing haemodialysis have been explored to a limited extent. In the study conducted by Ren et al. [236], the authors highlighted the potential of microbial markers as non-invasive diagnostic tools for CKD across various regions in China. They specifically characterised the GM in non-dialysis CKD patients, revealing a notable decrease in microbial diversity and significant alterations in comparison to the GM of healthy controls. Also, the authors displayed an enrichment of genera such as Klebsiella and Enterobacteriaceae, while *Blautia* and *Roseburia* were observed to be reduced in CKD patients [236].

(2) Differences in GM compositions in early-stage CKD patients compared to healthy controls. Hu et al. [237] noted differences in GM composition between patients in the early stages of CKD and healthy controls. They found that GM diversity was notably reduced in those with early-stage CKD compared to their healthy counterparts. At the genus level, *Ruminococcus* exhibited strong discriminatory capability in distinguishing early-stage CKD patients from healthy controls, while *Roseburia* proved to be a reliable indicator for identifying the healthy control group. Interestingly, a lower abundance of *Roseburia* has been reported in CKD patients from early stages, and a decrease is reported along with CKD progression, suggesting a possible role as a marker of GM dysbiosis [237,238].

(3) Association of GM composition and function with CKD severity. According to studies, the relative abundance of *Proteobacteria* increased gradually with the severity of CKD, with a mainly increased abundance of the family *Enterobacteriaceae* and the genera *Escherichia*, suggesting the enrichment of *Proteobacteria* as a potential microbial diagnostic marker of dysbiosis [239,240]. Additionally, most studies found that *Roseburia* levels were diminished in advanced CKD stages [238,241].

Overall, according to studies, patients with CKD exhibit a distinct profile of GM composition, characterised by variations in the abundance of specific genera and species; these alterations can potentially serve as valuable indicators in clinical models, enabling the differentiation between healthy patients with CKD.

### 6.2. Gut Dysbiosis in Kidney Stone (KS) Disease

Kidney stone (KS) disease is a prevalent condition, impacting approximately 10–15% of the worldwide population, and stands as the most prevalent urological ailment [242]. The primary type of kidney stone is calcium oxalate (76%), followed by hydroxyapatite (18%), uric acid (4.8%), struvite (0.9%), and brushite (0.9%) [243]. The prolonged presence of kidney stone disease can ultimately lead to diminished kidney function and additional health complications, while it has been observed that individuals with kidney stones are at an increased risk of developing transitional cell carcinoma (TCC), renal cell carcinoma (RCC), and kidney tumours [242].

Research on the gut microbiota’s role in KS disease primarily focuses on Oxalobacter formigenes, a Gram-negative bacterium known for its capacity to degrade oxalate [244]. It was previously suggested that a deficiency of *O. formigenes* in stool samples might be a risk factor for KS disease. However, clinical studies yielded inconclusive findings, as this bacterium was also found in samples from individuals with recurrent stone formation [245]. In a recent two-sample Mendelian randomization study, a causal link between the genus *Oxalobacter* and KS was not observed, suggesting that the association between the GM and KS is not solely dependent on the presence of the genus *Oxalobacter* or *O. formigenes* [246]. Recent research has attempted to evaluate the relationship between the GM and KS formation, shedding new light on the gut–kidney axis in nephrolithiasis.

The GM has been reported to demonstrate a significant role in both the pathogenesis and prevention of KS disease. The main findings regarding the involvement of the GM in KS disease include the following: 

(1) Alterations of GM composition in kidney stone patients compared to healthy controls [247,248,249]. In a recent—and the only available—meta-analysis conducted by Yuan et al. [247], encompassing 356 nephrolithiasis patients and 347 healthy subjects, the researchers found that individuals with kidney stones exhibited higher levels of *Bacteroides* and *Escherichia_Shigella* and a lower abundance of Prevotella_9. Additionally, the qualitative analysis demonstrated notable differences in beta-diversity between the two groups, while *Bacteroides*, *Phascolarctobacterium*, *Faecalibacterium*, *Flavobacterium*, *Akkermansia*, *Lactobacillus*, *Escherichia coli*, *Rhodobacter*, and *Gordonia* served as markers for the detection of KS.

(2) Involvement of the GM in KS formation [250,251]. In the study conducted by Deng et al. [250], the authors examined the associations between the composition and the abundance of the GM in 20 first-onset renal calculi patients, both before and after surgery. The OTU-based partial least squares discriminant analysis (PLS-DA) demonstrated distinctions between the RS1 (faecal samples taken before surgery) and RS2 (one-month post-surgery) groups. The examination of the GM using taxonomy-based comparisons showed differences in the GM composition, with the prevalence of *Enterobacteriales*, *Enterobacteriaceae*, *Gammaproteobacteria*, and *Escherichia* being higher in the RS2 group and the prevalence of *Pseudomonadaceae*, *Pseudomonadales*, and *Pseudomonas* being more abundant in the RS1 group. Furthermore, correlation analysis showed that an increased prevalence of *Enterobacteriaceae*, *Gammaproteobacteria*, and *Escherichia* was linked to decreased urea levels. Additionally, a decrease in creatinine level was correlated with a higher prevalence of *Escherichia*, highlighting the significant role of the GM in the formation of KS.

The data presented above strongly indicate the significant involvement of the GM in KS disease, and these discoveries have the potential to offer novel perspectives on the prevention, diagnosis, and treatment of renal stones.

### 6.3. Gut Dysbiosis in Renal Cell Carcinoma (RCC)

Renal cell carcinoma (RCC) is one of the most common types of cancer and originates in the renal parenchyma. The incidence of RCC varies widely from region to region, with the highest incidence reported in Western countries [252]. Worldwide, there is a global incidence of over 400,000 new cases of RCC, and more than 170,000 deaths occur annually [253]. The 2016 World Health Organization (WHO) classification identified distinct subtypes within RCC, each characterised by specific genetic and histopathological features. These include clear cell RCC (comprising 70–80% of cases), papillary RCC (10–15%), and chromophobe RCC (4–5%) [254]. Approximately two-thirds of RCC cases are localised, and the standard treatment involves complete surgical tumour resection [255]. Nevertheless, 30–40% of cases tend to progress to metastasis, irrespective of surgical intervention [255]. For early-stage renal cancer characterised as localised tumours, the 5-year relative survival rate is 92%, while the 5-year survival rate for patients with metastatic RCC is only 15% [255,256].

The exact role of the GM in the proliferation and differentiation of RCC is not fully understood. Nevertheless, it is suggested that dysbiosis could be a potential mechanism through which microbiota may contribute to the development and management of RCC. The main findings regarding the involvement of the GM in RCC include the following:

(1) Significant alterations in the GM distribution between the RCC patients and the healthy control group. In a recent study, the GM composition in 51 ccRCC patients and 40 healthy controls was analysed through 16S rRNA sequencing analysis [255]. According to the results, *Blautia*, *Streptococcus*, [Ruminococcus]_torques_group, *Romboutsia*, and [Eubacterium]_hallii_group were prevalent and positively associated with ccRCC. Furthermore, these microbial taxa demonstrated the ability to accurately differentiate between ccRCC patients and healthy individuals, suggesting their potential as biomarkers for ccRCC [255]. In the study of Yang et al. [257], the authors evaluated the possible association of specific members of the GM and their metabolites with clear cell renal cell carcinoma (ccRCC). The findings revealed notable variations in the relative abundances of 20 species between the RCC group and the control group. Among these, nine species were found to be more prevalent in the RCC group, including *Desulfovibrionaceae*, while 11 species were less prevalent, including four types of *Lactobacillus*.

(2) Contribution of GM dysbiosis to the formation of KS, subsequently leading to an increased risk of RCC. The data presented in the previous section indicate the significant involvement of the GM in KS disease, mainly in the formation of KS. The review study by Gupta et al. [242] shed light on a higher risk of kidney cancer in patients with recurrent KS compared to subjects with no history of KS disease.

Furthermore, a recent review by Yang et al. [258], investigated the influence of the GM on RCC treatment and suggested that the GM composition could potentially serve as a predictive marker for the potential effectiveness of immune checkpoint inhibitors in RCC patients. The review also provided evidence supporting the idea that modulating the abundance and distribution of the GM can enhance the therapeutic impact of drugs, indicating that microbiota could be a promising adjunctive treatment for RCC.

### 6.4. The Role of Prebiotics in Kidney Diseases

Numerous clinical trials and experimental investigations in CKD have demonstrated that the use of prebiotics, probiotics, and synbiotics can effectively decrease the levels of uraemic toxins like p-cresyl and indoxyl sulphates, as well as reduce inflammatory markers [226,235,259]. Additionally, these interventions have been shown to attenuate disruption of the tight junctions in the colonic epithelium, leading to significant enhancements in conditions such as endotoxemia, blood urea nitrogen levels, and overall quality of life [235,259].

GM manipulation with prebiotic, probiotic, and synbiotic supplementation has emerged as a potential therapeutic intervention in kidney diseases, but the evidence for the effects of prebiotics, especially in human studies, is scarce [260]. In a recent review paper conducted by McFarlane et al. [261], it was demonstrated that there was low certainty about the effect of these agents owing to the varying results. Limited clinical trials have investigated the use of prebiotics for GM manipulation in CKD, while in KS disease and RCC, there have been no clinical studies, making this an attractive therapeutic option to explore (Table 2). In the study of Laffin et al. [83], a double-blind, parallel, randomised, placebo-controlled trial was performed comparing dietary supplementation of high-amylose maize-resistant starch type 2 (HAM-RS2) with a placebo in patients with end-stage CKD. According to the results, prebiotic supplementation resulted in a decrease in systemic inflammation and an increase in *Faecalibacterium* [83]. Furthermore, the effects of prebiotic lactulose supplementation on faecal microbiota in patients with stages 3 and 4 CKD were recently examined [84]. An 8-week prebiotic intervention resulted in a significant decrease in creatinine and an increase in faecal *Bifidobacteria* and *Lactobacillus* counts in CKD patients. Additionally, in the study of Biruete et al. [85], the impact of a 4-week supplementation of inulin on the GM composition and microbial metabolites of patients undergoing haemodialysis was examined. Inulin supplementation increased the relative abundance of the phylum *Verrucomicrobia* and its genus *Akkermansia*. Recently, the effect of β-glucan on kidney function, uremic toxins, and the GM was examined in stage 3 to 5 CKD participants [86]. Supplementation of β-glucan fibre resulted in reduced plasma levels of the free fraction of colon-derived uremic toxins over the 14-week study period. Additionally, prebiotic supplementation resulted in a high prevalence of Bacteroides 2 in the CKD population.

In summary, GM modulation and improvement of gut dysbiosis in kidney diseases by prebiotics is an area of ongoing research. Large numbers of rigorous clinical trials are needed to further confirm the effects and safety of prebiotics and optimise the methods and durations of treatment.

**Table 6:** Main dysbiotic events that occur in GM during the onset and progression of kidney diseases.

Kidney Disease	Main Dysbiotic Events	Reference
Chronic kidney disease (CKD)	- Patients with CDK display increased gut levels of Lachnospiraceae, Enterobacteriaceae, Enterococci, Clostridium perfringes, and certain Ruminococcaceae, and decreased levels of Prevotellaceae, Bacteroidaceae, and in particular, Lactobacillus and Bifdobacterium species - Ruminococcus exhibits strong discriminatory capability in distinguishing early-stage CKD patients from healthy controls - Relative abundance of Proteobacteria increased gradually with the severity of CKD	[233,234,239,240]
Kidney stone (KS) disease	- Individuals with KS exhibited higher levels of *Bacteroides* and *Escherichia_Shigella* and a lower abundance of *Prevotella_9* - *Bacteroides*, *Phascolarctobacterium*, *Faecalibacterium*, *Flavobacterium*, *Akkermansia*, *Lactobacillus*, *Escherichia coli*, *Rhodobacter,* and *Gordonia* served as markers for the detection of KS - Involvement of the GM in KS formation	[247,250,251]
Renal cell carcinoma (RCC)	- Significant alterations in the GM distribution between the RCC patients and the healthy controls - Contribution of GM dysbiosis to the formation of kidney stones, subsequently leading to an increased risk of RCC	[242,255,257]

## 7. Limitations of Modulating Gut Microbiota via Prebiotics

There are several limitations regarding the effects of modulating the GM through prebiotics [262], including the following: (1) The limitation of efficacy. The GM displays significant diversity in both taxonomic composition and functions, irrespective of an individual’s health condition [263]. This inter-individual variability that characterises GM profiles among subjects could potentially compromise the efficacy of prebiotics [262] due to significant variations in individual responses to specific prebiotics. It is worth noting that approximately 20% of the aforementioned interpersonal variability in microbial signatures can be attributed to environmental factors associated with diet and lifestyle [263]. Consequently, reproducing results becomes a challenge. Moreover, the improvement of culture techniques and high-throughput sequencing technologies will play a role in the identification of specific microbial species and their roles, as well as the identification of the interaction between the GM and various organs. (2) Reduction of efficiency. Prebiotics’ efficacy may be reduced due to their transient lifespan as they pass through the gastrointestinal tract. (3) Prebiotics should be consumed or administered on a regular basis in order to confer a significant effect. Furthermore, while prebiotics stimulate the growth of specific beneficial bacterial populations, this growth may be temporary, limiting the host’s ability to gain health benefits [262]. Additionally, the side effects of prebiotics are caused by their osmotic actions. Prebiotic consumption can lead to osmotic diarrhoea, bloating, cramping, and flatulence, while prebiotics with shorter chain length may have more negative effects [264]. Overall, designing customised, population-based, specific prebiotics based on the GM specific to each community may eventually contribute to health benefits.

## 8. Conclusions

In recent times, there has been a significant focus on the importance of the GM in maintaining host well-being. Scientific research has revealed connections between imbalances in the GM and diseases that extend beyond gut-related issues, affecting organs such as the brain, liver, lungs, heart, and more (Figure 1). Consequently, the question arises: do these alterations in the GM contribute to the diseases, or do they merely reflect the diseases’ status? Often, the development of diseases affecting distant organs has been linked to gastrointestinal discomfort or disorders. The communication and interaction between the GM and distant organs are gaining increasing recognition, with ongoing efforts to gradually unravel the intricacies of host–microbiome interactions, which have a profound impact on overall host health.

Current research is focused on identifying microbiome signatures as potential indicators of various health conditions. Understanding the gut–organ axis and its interactions with distant organs can assist in the development of strategies for disease prevention and therapy. While some studies have suggested the potential therapeutic use of prebiotics for various diseases, further investigation is needed to understand their long-term effects, their impact on the host–microbiome balance, and their effects on GM composition. Clinical trials with standardised prebiotic dosages, extended administration durations, and regular follow-ups are essential to confirm the efficacy of prebiotics in manipulating the gut–organ axis.

## Data Availability

No new data were created or analyzed in this study. Data sharing is not applicable to this article.
